# A post-transcriptional regulatory landscape of aging in the female mouse hippocampus

**DOI:** 10.3389/fnagi.2023.1119873

**Published:** 2023-03-24

**Authors:** Raphaelle Winsky-Sommerer, Helen A. King, Valentina Iadevaia, Carla Möller-Levet, André P. Gerber

**Affiliations:** Faculty of Health and Medical Sciences, School of Biosciences and Medicine, University of Surrey, Guildford, Surrey, United Kingdom

**Keywords:** brain, translatome, transcriptome, alternative splicing, RNA-binding protein, Polysome analysis, mitochondria, polyadenylation

## Abstract

Aging is associated with substantial physiological changes and constitutes a major risk factor for neurological disorders including dementia. Alterations in gene expression upon aging have been extensively studied; however, an in-depth characterization of post-transcriptional regulatory events remains elusive. Here, we profiled the age-related changes of the transcriptome and translatome in the female mouse hippocampus by RNA sequencing of total RNA and polysome preparations at four ages (3-, 6-, 12-, 20-month-old); and we implemented a variety of bioinformatics approaches to unravel alterations in transcript abundance, alternative splicing, and polyadenylation site selection. We observed mostly well-coordinated transcriptome and translatome expression signatures across age including upregulation of transcripts related to immune system processes and neuroinflammation, though transcripts encoding ribonucleoproteins or associated with mitochondrial functions, calcium signaling and the cell-cycle displayed substantial discordant profiles, suggesting translational control associated with age-related deficits in hippocampal-dependent behavior. By contrast, alternative splicing was less preserved, increased with age and was associated with distinct functionally-related transcripts encoding proteins acting at synapses/dendrites, RNA-binding proteins; thereby predicting regulatory roles for RBM3 and CIRBP. Only minor changes in polyadenylation site selection were identified, indicating pivotal 3′-end selection in young adults compared to older groups. Overall, our study provides a comprehensive resource of age-associated post-transcriptional regulatory events in the mouse hippocampus, enabling further examination of the molecular features underlying age-associated neurological diseases.

## Introduction

Aging is associated with a plethora of physiological changes linked to a wide spectrum of biological functions (Gorgoulis et al., 2019; Schaum et al., 2020) and is a major risk factor for several disorders including dementia (Hou et al., 2019; Vincent and Yaghootkar, 2020). Brain aging may be associated with mild cognitive impairment and neurodegenerative disorders, the incidence of which is rising world-wide. To identify the molecular processes associated with physiological and pathological aging, many preclinical studies focused on characterizing the effects of aging on gene expression, revealing that age-related alterations in gene expression are extensive, tissue- and sex-specific (Berchtold et al., 2008; Tower, 2017; Schaum et al., 2020). Despite the well documented age-related decline in immune function, aging is generally associated with an upregulation of immune- and inflammation-associated processes. At the same time, mitochondrial function decreases substantially with age, which correlates with the downregulation of mRNAs coding for mitochondrial proteins. Additional gene expression features of aging concern a decrease of mRNAs coding for ribosomal proteins, a reduction in growth factor signaling and possibly the constitutive response to stress and DNA damage as well as the dysregulation of transcription and RNA processing (Bishop et al., 2010; Frenk and Houseley, 2018). Importantly, the effects of aging on the brain’s transcriptome profiles are mostly concordant in humans and mice (Hargis and Blalock, 2017).

The hippocampus is a key brain region involved in learning, memory consolidation and retrieval, as well as forgetting (Bartsch and Wulff, 2015; Yonelinas et al., 2019). Thus, it has been extensively studied in the context of aging and cognitive decline. Moreover, as part of the limbic system, it also plays a role in the regulation of emotions and is associated with the development of neuropsychiatric symptoms observed in dementia. Hippocampal neuroplasticity and neurogenesis are susceptible to adverse conditions such as stress, ischemia, neurodegeneration, and aging, the latter two being associated with the accumulation of proteins such as tau or β-amyloid peptide (Fjell et al., 2014; Bettio et al., 2017; Navarro-Sanchis et al., 2017). Microarray and RNA sequencing studies identified age-related changes in the expression and alternative splicing of genes contributing to the immune response, inflammation, as well as protein processing, oxidative stress, and synaptic plasticity in the rodent hippocampus (Verbitsky et al., 2004; Xu et al., 2007; Stilling et al., 2014; Pardo et al., 2017). Some of these changes were observed in parallel to the decline in spatial memory or novel object recognition memory (Verbitsky et al., 2004; Pardo et al., 2017). In addition, sex appeared to have a marked effect on the hippocampal transcriptome in middle-aged and old mice (Xu et al., 2007; Berchtold et al., 2008; Mangold et al., 2017), and similar sex differences have been observed in the human hippocampus (Guebel and Torres, 2016).

While characterizing the genome-wide expression of transcripts has provided valuable insights into the impact of aging on cellular and molecular functions, translation substantially contributes to the regulation of protein-coding gene expression (Sonenberg and Hinnebusch, 2009; Woodward and Shirokikh, 2021). As such, translatome analysis enables to monitor translational regulation of gene expression, solely by focusing on active transcripts associated with ribosomes (King and Gerber, 2016). A recent study combining RNA-sequencing and ribosome profiling showed decreased translation of transcripts involved in protein synthesis machinery with aging in the mouse liver and kidney, as well as alterations in the distribution of ribosome coverage (Anisimova et al., 2020).

In this study, we compared age-related changes in the translatome and transcriptome in the hippocampus of female C57BL/6 mice, and comprehensively monitored differences in gene expression, alternative splicing, and polyadenylation site selection. As most previous age-related studies were conducted with male mice, we thought to perform this analysis with female mice which are commonly underrepresented in mice studies. We also did not simply contrast young and older mice but instead studied mice at four ages, i.e., 3-, 6-, 12-, and 20-month-old, revealing age-related changes in molecular processes that are highly time-dependent, and suggesting distinct subsets of transcripts prone to translational regulation. Alternative splicing was rather divergent between the transcriptome and translatome, became slightly increased with age and affected functionally related sets of genes different to those with changed expression. Conversely, we could not identify age-dependent alterations of poly (A) site selection. Overall, this study revealed significant effects of aging at the level of translational efficiency and alternative RNA splicing, providing a unique resource for further study of mouse hippocampi function and the associated cognitive impairments associated with aging.

## Results

**Figure 1:**
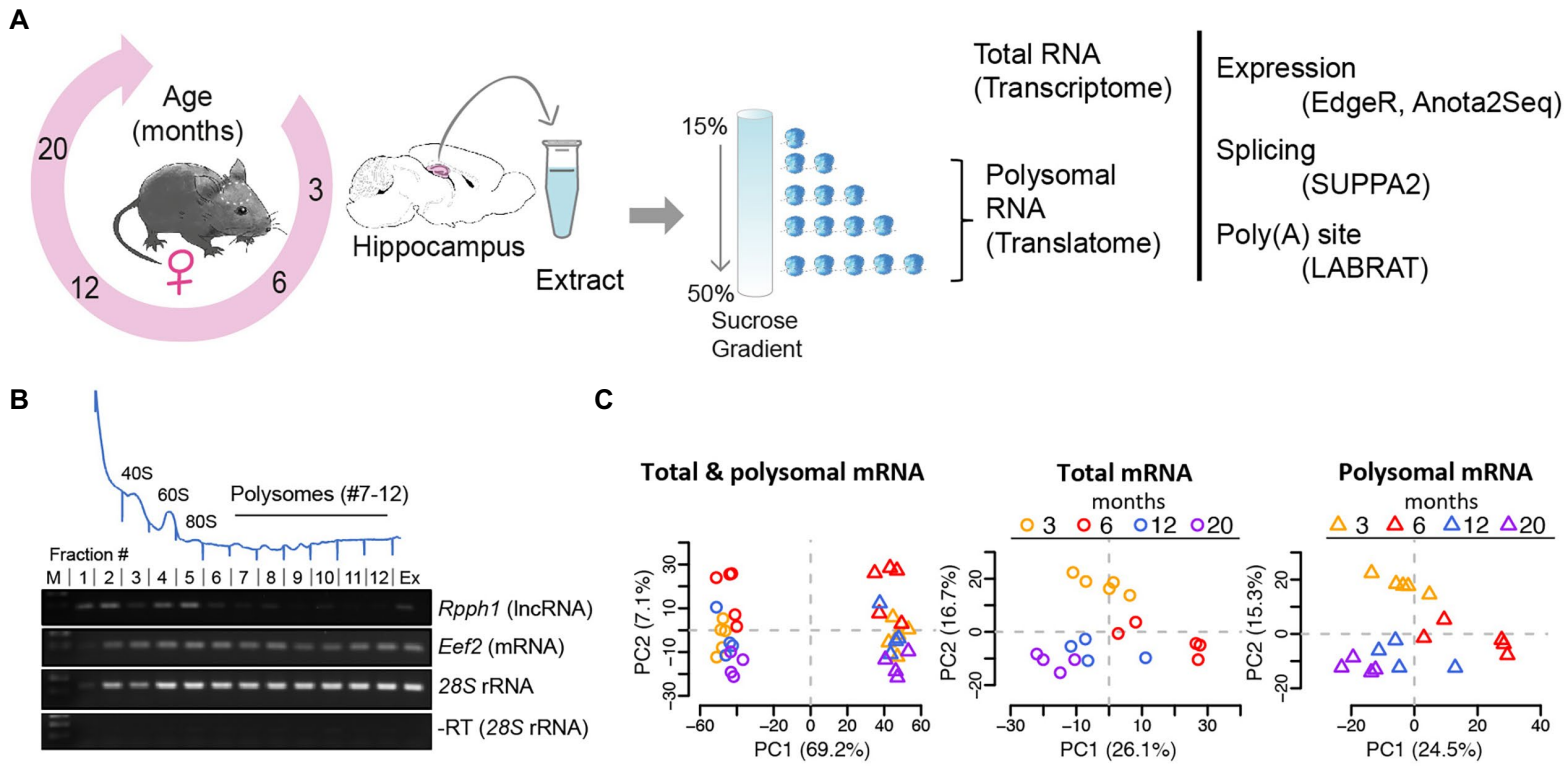
Analysis of mRNA translation by sucrose density gradients and RNA-sequencing. **(A)** Experimental scheme: hippocampi from female mice aged 3, 6, 12, and 20  months were collected, extracts prepared and fractionated on sucrose gradients. Total RNA samples and polysomal RNA samples were sequenced, representing the transcriptome and translatome, respectively for further analysis **(B)** Representative absorbance profile at 254 nm across a sucrose gradient from hippocampi (on top). Positions of the 40S and 60S ribosomal subunits, 80S monosomes, and polysomes are indicated. Fractions 7–12 of the polysomes were collected for RNA-seq representing the translatome. Bottom: RT-PCR with isolated RNA from fractions #1–12 of the sucrose gradient. Extract (Ex) refers to total RNA isolation corresponding to the transcriptome; (M) molecular weight marker. *Rpph1*: long non-coding RNA (lncRNA) not expected to be translated; *Eef2*: eukaryotic elongation factor 2 mRNA and 28S rRNAs are expected to be present in all fractions including polysomes. A negative control PCR reaction without RT for 28S rRNA is shown at the bottom. **(C)** PCA after outlier removal and batch correction of all samples (*n* = 36; left column), total mRNA samples (*n* = 18, circles), and polysomal mRNA samples (*n* = 18, triangles). Samples are colored by age. The component percentages are indicated in brackets.

To monitor the changes of the transcriptome and translatome during aging, we collected hippocampi from four age groups of female C57BL/6 mice encompassing mature adulthood (3- and 6-month-old) as well as middle- and old- age (12- and 20-month-old), equivalent to ~20–30, ~40+, and ~65 years in humans (Flurkey et al., 2007). Total RNA was extracted to monitor global changes of the transcriptome, while sucrose density fractionation was performed to collect polysomes, which represents transcripts of the translatome (considering fractions 7–12 of the gradient; Figures 1A,B; polysomal profiles of all samples are displayed in Supplementary Figure 1). Polyadenylated (poly (A)) RNA was further selected from matched samples and subjected to RNA sequencing. Principal component analysis (PCA) and Pearson correlation of TMM normalized log_2_ CPM were used to evaluate batch effects, outliers, and sample similarity. Batch correction/outlier analysis was performed (see Materials and Methods) and the processed data showed good segregation between the transcriptome and translatome and different ages (Figure 1C, PCA analysis of original and batch-corrected data is shown in Supplementary Figure 2).

### Differential expression analysis reveals convergent age-related expression trajectories in the hippocampal transcriptome and translatome

**Figure 2:**
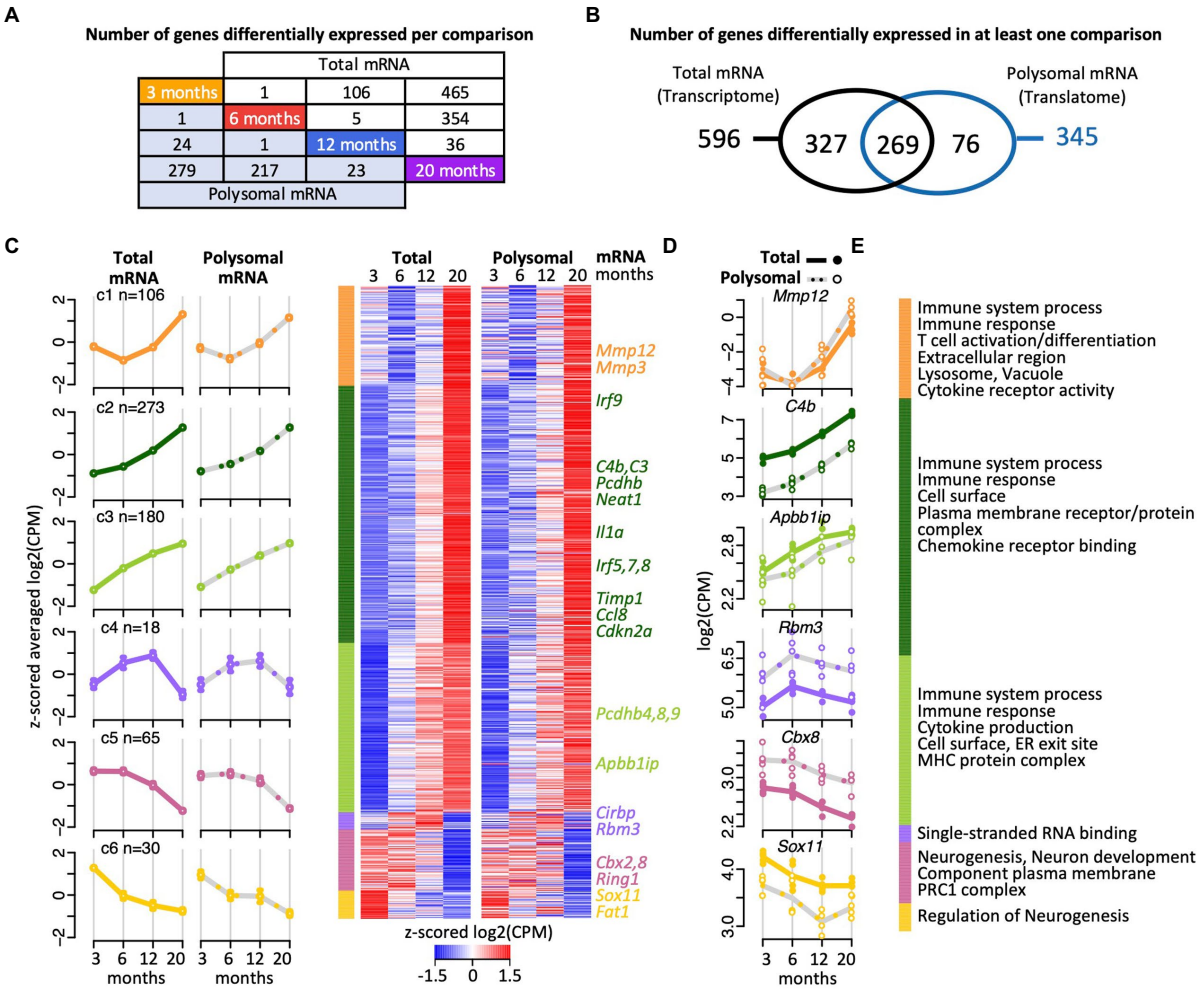
Gene expression trajectories in the transcriptome and translatome across four age groups identified with edgeR. **(A)** Scheme depicting the number DEGs in the transcriptome (total RNA) and translatome (polysomal RNA) across all pairwise time comparisons. **(B)** Venn diagram representing overlap of DEGs in the transcriptome and translatome. **(C)** Cluster heatmap and averaged temporal profiles of expression for the 672 DEGs identified in the transcriptome and translatome. The log_2_(CPM) values for each gene in a single age group were averaged to generate z-scored mean profiles, and six clusters (C1–C6; marked in different colors) were identified with the Bayesian Index Criterion based on the total RNA (transcriptome) data. The corresponding polysomal RNA (translatome) data are displayed to the right. The average profiles for each cluster are shown to the left with a continuous line for total RNA, a gray dashed line for polysomal RNA, and error bars showing 95% confidence intervals (CI). The number of genes within each cluster is indicated (*n*). **(D)** Expression levels (log_2_(cpm)) of selected genes. Total mRNA; solid line displays the mean and closed circle refer to individual samples. Polysomal mRNA; gray dotted line is the mean and open circles show individual values. **(E)** Enriched Gene Ontology (GO) terms for each cluster. The dataset with functional annotations is provided in Supplementary Table 2.

We first used edgeR, a classical Bioconductor package to perform RNA-seq gene expression analysis based on count-based data (Robinson et al., 2010, McCarthy et al., 2012; processed edgeR data as well as raw count data is provided in Supplementary Table 1). Only a small fraction of all genes (3.9%) across the transcriptome and the translatome significantly changed in relative expression upon aging in at least one pairwise time comparison (Figures 2A,B; 672 out of 16,801 genes with Benjamini Hochberg (BH) corrected *p* < 0.05 and abs (FC) > log_2_ (1.2); list of DEGs provided in Supplementary Table 2; the *p*-value distribution is displayed in the Supplementary Figure 3). The majority of these differentially expressed genes (DEGs) were up-regulated (83%, *n* = 559), which is consistent with previous transcriptome profiling in the mouse hippocampus (Swindell et al., 2012; Gatta et al., 2014; Stilling et al., 2014). While 96% (*n* = 646) of those DEGs coded for proteins, the remaining included 25 ncRNA genes (e.g., *Neat1*) and one pseudogene (*3000002C10Rik*; glyceraldehyde-3-phosphate dehydrogenase pseudogene). *Neat1* was previously seen to be increasingly expressed during aging in the mouse hippocampus (Stilling et al., 2014) and in the human brain (Butler et al., 2019); and the manipulation of *Neat1* expression in the hippocampal CA1 region was recently shown to modulate hippocampus-dependent memory assessed by contextual fear conditioning in mice (Butler et al., 2019). Thus, along the emerging functional relevance of age-regulated ncRNAs (Szafranski et al., 2015; Barter and Foster, 2018), our results suggest that possibly dozens of lncRNAs are implicated in hippocampal aging and the associated cognitive functions. We wish to note that besides lncRNA, RNA-seq reads have also been mapped to several small non-coding RNAs (e.g., the snRNA *Rnu7*). Those instances would need further investigation as they may raise from reads of overlapping ORFs, such as *Grcc10*, a gene rich cluster overlapping with *Rnu7*.

Functional enrichment analysis of the 646 protein coding gene transcripts revealed that 41% of them act in immune system processes (228 of the total of 553 annotated Entrez IDs for Gene Ontology (GO) Biological Process, FDR = 0), with 161 genes associated to cell-surface receptor signaling pathways (FDR = 0), such as the Toll-like receptor signaling pathway (e.g., 5 of the 6 genes acting in the toll-like receptor 7 signaling pathway; *p* < 5 × 10^−7^, FDR < 1.7 × 10^−5^), and/or are related to infection and inflammatory responses; and acting in the response to external biotic stimulus (110 genes, FDR = 0), leading to leukocyte activation (98 genes, FDR = 0) and cytokine production (94 genes, FDR = 0; Supplementary Table 2). We wish to note that likewise functional enrichment analysis of the 76 DEGs identified exclusively in the translatome (Figure 2B) did not reveal any striking differences to these functional themes (e.g., immune system processes; FDR < 2 × 10^−3^). Overall, these data recapitulate previous observations regarding the strong activation of immune and inflammatory responses during aging in the mouse and human hippocampus (e.g., Xu et al., 2007; Hargis and Blalock, 2017; Ianov et al., 2017; Frenk and Houseley, 2018; González-Velasco et al., 2020).

We next clustered the expression profiles of the 672 DEGs to identify groups of genes with similar expression trajectories across the four age groups (see Materials and methods). Six clusters were identified. They showed remarkably similar changes in expression at the transcriptome and translatome levels suggesting coherent responses (Figures 2C,D; Supplementary Table 2). The three most prominent clusters (C1–C3) included 83% of the DEGs (*n* = 559) with increased expression at the endpoint of 20 months (Figures 2C,D). Besides genes associated with immune system processes and inflammatory responses, these clusters included several cell senescence markers, such as *Mmp3*, *Mmp12, Cdkn2a*, *Ccl8*, *Il1a*, *Timp1,* which are known to be increasingly expressed in various tissues of aged animals (Figures 2E) (Hudgins et al., 2018; Gorgoulis et al., 2019). Furthermore, we found that Irf1-q6 transcription factor binding sites were particularly overrepresented among the genes in C2 and C3 clusters (i.e., C3: 19 of 116 annotated genes; FDR < 1.3 × 10^−5^; Supplementary Table 2). These represent putative binding sites for interferon regulatory factors (IRFs), which is a group of transcription factors (TFs) involved in modulation of cell growth, differentiation, apoptosis, and immune system activity. Finally, the C2 and C3 clusters also contained several genes coding for protocadherins (C2: *Pcdhb1,2,3,5,6,14*; C3: *Pcdhb4,8,9*), which code for cell-adhesion proteins with functions in the immune response and in the plasticity of hippocampal circuits (Kim et al., 2010; Keeler et al., 2015); and were previously shown to be upregulated in astrocytes of aged mice (Boisvert et al., 2018). Besides those stably progressing profiling, 18 genes that grouped in the smallest cluster (C4) showed a gradual increase in expression up to 12-month-old, followed by a drastic decline at 20 months (Figures 2C-E). Here, four of the 14 protein coding genes encode mRNA-binding proteins (mRBPs), with roles in splicing (*Rbm11*), mRNA decay/translation (*Cirbp*), translation regulation (*Rbm3*), or interacting with mRNA 3′-end formation/polyadenylation complex (zinc finger CCCH type containing 6 (*Zc3h6*) protein). The expression trajectories for these genes suggest dynamic post-transcriptional control, in accordance with the emerging role of RBPs in brain aging and cellular senescence (Wei et al., 2015; Dong et al., 2018; D'Amico et al., 2019). Only 95 genes (14%) displayed a gradual decline in expression with age and were grouped into two clusters (C5, C6; Figures 2C,D). These clusters were overrepresented for genes related to neurogenesis (29 of 82 mapped protein coding genes; FDR < 3 × 10^−5^), neuronal differentiation (25 genes; FDR < 1.5 × 10^−4^) and development (21 genes; FDR < 10^−3^), many of the encoded proteins localized to the plasma membrane (25 genes; FDR < 2.2 × 10^−6^) (Figure 2E). Interestingly, the C5 cluster contained three components of the polycomb repressive complex 1 (PRC1; namely *Cbx2*, *Cbx8*, *Ring1*; FDR < 4 × 10^−3^), a multiprotein complex mediating mono-ubiquitination of lysine residues of histone H2A in mammals and required for long-term maintenance of transcriptionally repressed states and chromatin remodeling (Shao et al., 1999). PRC1 regulates *Cdkn2A*, whose expression increases in mammalian cells with senescence and age (identified in C2 cluster). Since Polycomb (PcG) proteins maintain chromatin in ‘off’ states, thereby preventing expression, the reduced expression of PRC1 components is consistent with the observed increased levels *Cckn2A* transcripts with age (O'Sullivan and Karlseder, 2012).

In conclusion, the six concordant age-related trajectories in transcript abundance emphasize previous findings for age-related alterations of genes associated with the immune system, inflammation, and neurogenesis, thereby validating our experimental approach. In addition, distinct temporal profiles, such as the progressive increase in expression trajectory from 3 month- to 12- month-old followed by a sharp decline were identified and include several RBPs suggestive of modulation of post-transcriptional control in older mice, while the observed repression of particular ‘neuron-related’ genes is reminiscent to the decline of associated brain functions with age.

### Analysis of translational activity highlights divergence between the transcriptome and translatome for discrete subsets of genes

**Figure 3:**
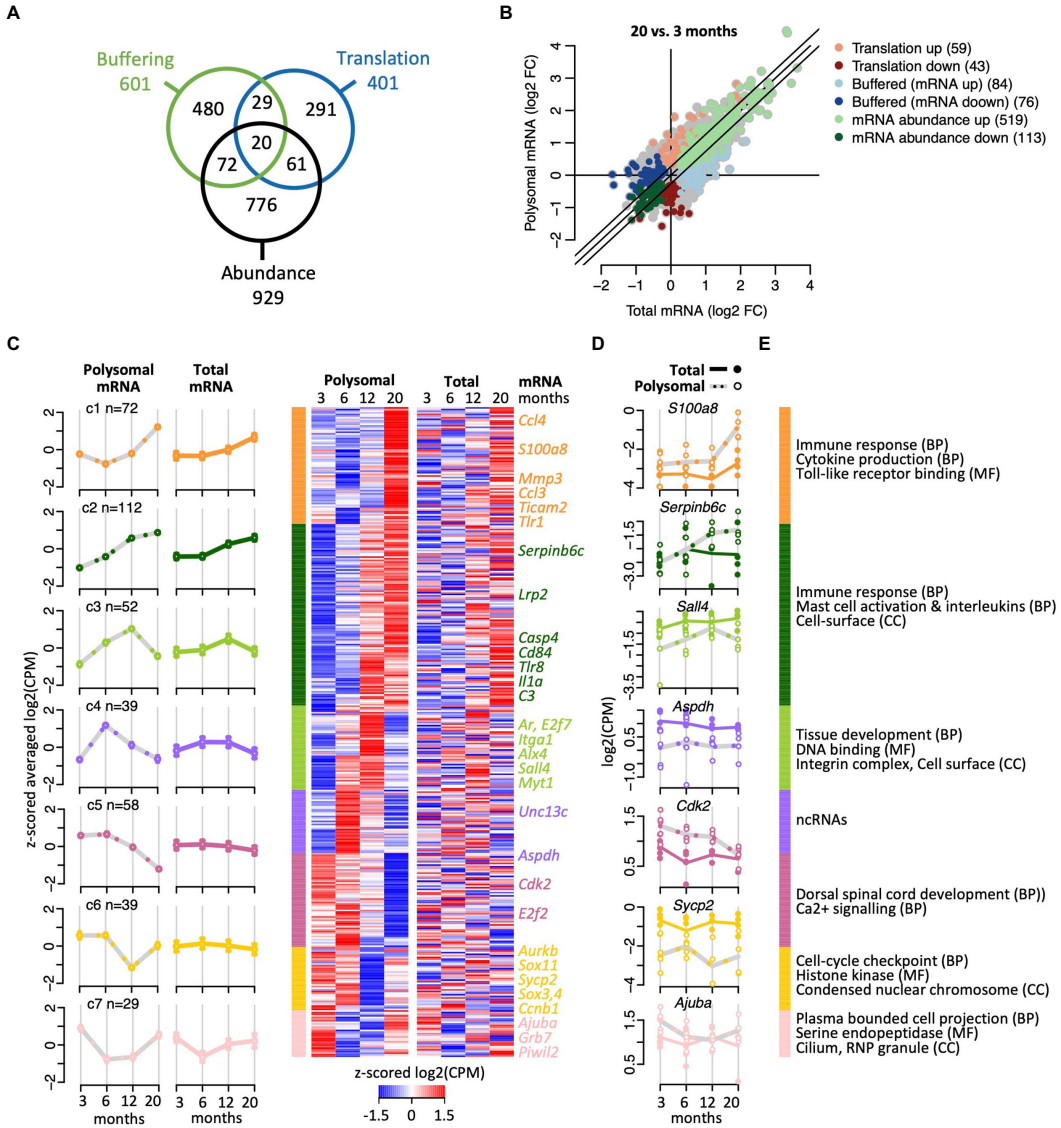
Translational efficiency analysis identifying age-related changes of mRNA expression. **(A)** Venn diagram shows overlap of numbers of transcripts identified with anota2seq across the indicated three regulatory modes. **(B)** Fold-changes (FC) in total mRNA (*x*-axis) and polysomal mRNA levels (*y-*axis) of 20-month-old vs. 6-month-old mice. Colors depict genes allocated to the three regulatory modes. Number of genes is indicated within brackets. **(C)** Cluster heatmap and averaged temporal profiles of genes displaying alteration in the ‘translation’ regulatory mode during aging (*n* = 401 genes). Clustering was based on the polysomal RNA (translatome) profiles; corresponding total RNA (transcriptome) data is displayed to the right. Average profiles for each cluster are shown to the left with a continuous line for total RNA, a gray dashed line for polysomal RNA, and error bars marking 95% CI. **(D)** Expression levels (log_2_(cpm)) of selected genes. Total mRNA; solid line displays the mean and closed circles refer to individual samples. Polysomal mRNA; gray dotted line is the mean and open circles show individual values. **(E)** GO terms enriched with each cluster. GO categories: BP, biological process, CC, cellular compartment, MF, molecular function. The complete dataset including GO annotations is given in Supplementary Table 1.

To further characterize the effects of aging on translation, we next applied analysis of translational activity (anota2seq; Oertlin et al., 2019). Anota2seq compares expression data originating from translated mRNA to data from matched total mRNA, enabling identification of (i) translated mRNA that are not paralleled by corresponding changes in total mRNA (interpreted as changes in translation efficiencies impacting protein levels; referred to as ‘translation’ group), (ii) congruent changes in total and translated mRNA (interpreted as changes in transcription and/or mRNA stability; ‘abundance’ group); and (iii) changes in total mRNA not paralleled by corresponding alterations in translated mRNA (interpreted as translational buffering; ‘buffering’ group; Oertlin et al., 2019). Applying the same criteria as for the differential expression analysis with edgeR (BH corrected *p* < 0.05; abs (FC) > log_2_ (1.2)) and investigating all paired-age comparisons, anota2seq identified 1,729 DEGs, showing a strong overlap (82%; *n* = 554) with the previously identified 672 DEGs using edgeR (the processed annota2Seq data is given in Supplementary Table 3). 1,489 of those 1,729 genes code for proteins (86%), 194 for lncRNAs (11.2%; including *Neat1*), 26 represent pseudogenes (1.5%), and 20 bear unknown or other functions (1.16%). As expected, the 1,489 protein coding genes were strongly enriched for immune response (249 genes, FDR = 0) and cell-surface receptor signaling pathways (292 genes, FDR = 0). Further noteworthy is the consistent allocation of genes coding for proteins acting at the cell-periphery (i.e., plasma membrane, cell-surface, and extracellular region) and the cytoskeleton (173 genes, FDR = 7 × 10^−4^), including 10 components of the kinesin complex (FDR = 0.016). While most of the selected DEGs were allocated to the ‘abundance’ regulatory mode (*n* = 929), 601 DEGs were allocated to the translational ‘buffering’ mode; and 401 genes were classified (in at least one of the paired age comparisons) in the ‘translation’ mode associated with significant changes in translational efficiencies (Figure 3A). Notably, only a minor fraction of genes (182 genes, <5%) were selected in at least two different regulatory modes, indicating consistent association of most transcripts with a particular regulatory mode over time.

Considering all age-comparisons, most changes in gene expression were identified comparing 20- vs. 6-month-old mice (894 genes; Figure 3B), while 20 vs. 12 months identified the least (176 genes; all pairwise time comparisons are displayed in Supplementary Figure 4). Furthermore, 6- vs. 3-month-old female mice revealed not only most changes (405 genes) among the “neighbored” time comparison but also the largest fraction of down-regulated genes (229 of 405 genes, 57%). Therefore, as the number of DEGs progressively declined at later age comparisons (i.e., 207 and 176 genes comparing 12 vs. 6 and 20 vs. 12 months aged mice, respectively), most changes in gene expression seem to occur during maturation from young adulthood to middle age and not at progressed age.

We next clustered the genes within each regulatory mode to identify common time-dependent expression trajectories. Thereby, seven temporal profiles were identified among transcripts allocated to the ‘translation’ regulatory mode prone to translational regulation (Figure 3C; complete dataset with GO annotation is given in Supplementary Table 4). Herein, the largest clusters (C1 and C2, *n* = 184) contained genes that were increasingly expressed with age and code for proteins acting in the immune response and inflammation, with many of them located on the cell surface or being secreted (Figures 3C-E). Interestingly, the other five clusters comprised genes undergoing peak expression at defined time-points: for 52 genes (C3) expression peaked at 12 months, encompassing 14 genes related to tissue development (*p* < 7.8 × 10^−6^) and ‘DNA binding’ (10 genes, *p* < 0.003; e.g., *Alx, Ar, E2f7, Nlox3-1, Sall4, Zfp628*), and two components of the integrin complex (*Itga1, Itgbl1*). For 39 genes (C4), the expression peaked in the translatome at 6-month, while three additional profiles suggested pronounced translational repression at 20 months (C5; 58 genes), 12 months (C6; 39 genes) or extended repression between 6 and 12 months (C7; 29 genes). Since those clusters (C3–C7) contained relatively few transcripts, no functional associations could be identified among those with an FDR < 0.05. Nevertheless, C4 contained a high proportion of known or predicted (long) ncRNAs (14 out of 39 genes, 36%), many of them expressed in the brain. The function of those lncRNAs in association with polysomes is not known, but it could relate to translational regulatory functions or encoding small peptides (Minati et al., 2021; Duffy et al., 2022). C5 and C6 clusters contained protein coding genes involved in calcium signaling (e.g., *Agtr1a*), cell-cycle checkpoint (*Cck2, E2f2*, *Sox11, Sox4, Aurkb,* and *C1*) and a component of synaptonemal complex protein 2 (*Sycp2*); and the C7 cluster includes protein coding genes acting at the cilium and axomene (e.g., *Mak, Ccdc114, Cfap73*), and ribonucleoprotein granules (e.g., *AjubA*, *Grb7*, *Piwil2*). Overall, these data indicate time-dependent translational regulation of mRNA subsets, possibly fine-tuning physiological alterations occurring at specific ages.

Likewise clustering analysis identified five temporal profiles for the 929 genes altering their “abundance” similarly in the transcriptome and translatome, and six clusters grouping the 601 DEGs identified in the ‘buffering’ regulatory mode (i.e., where significant changes in transcriptome are not reflected at the translatome level), (Supplementary Figure 5; data in Supplementary Table 4). Most genes of the ‘abundance’ group showed a highly coordinated gradual increase in expression with age (Supplementary Figure 5A; clusters C2, C3; 554 genes, 59.6%) and were mostly allocated to the immune response, the cell-surface and plasma membrane reminiscent of the differential gene expression analysis with edgeR. Conversely, the expression of 199 genes (clusters C4 and C5) was significantly reduced in aged animals at 20 months, including 26 genes involved in neurogenesis (C5, *p* < 8.5 × 10^−5^, FDR < 0.08), such as *Ndnf* and *Draxin* as well as 9 genes coding for proteins associated with the mitotic spindle like cyclin B1 (*Ccnb1*). Remarkably, 176 genes showed a pronounced V-shape expression, with substantially lower expression at 6 months compared to 3 months but recovering the expression after 12 month and further increasing toward 20 months (C1 cluster in Supplementary Figure 5A). The genes in this cluster coded for proteins involved in transmembrane transport (34 genes, FDR < 1.8 × 10^−4^), especially ion transporters (33 genes, FDR < 9 × 10^−4^), such as copper/iron ion importers (i.e., *Steap1/2/3*; Knutson, 2007) and several solute carriers (Slc) that belong to the largest family of transmembrane transporters, facilitating the exchange of ions, nutrients, metabolites and drugs across biological membranes (i.e., *Slc12a2, Slc16a6, Slc16a8, Slc31a1, Slc37a2, Slc24a5, Slc2a12, Slc39a4*). The temporal profiles of the 601 DEGs identified in the ‘buffering’ regulatory mode showed greater diversity (Supplementary Figure 5B). Only a minor fraction of genes (265 genes, 44%) steadily increased expression with age, mainly including genes associated with immune and inflammatory response, while fluctuating or V-shape expression concerned most genes. The latter group included genes coding for cellular compartments such as the cilium, cytoskeleton, and the chromosome (Supplementary Figure 5B; Supplementary Table 4). In conclusion, substantial fluctuations in the temporal expression were observed among genes selected in the ‘translation’ and ‘buffering’ mode, while most genes steadily changed expression unidirectionally with time in the ‘abundance’ group and reflect coordinate changes in the transcriptome and translatome.

### Gene set enrichment analysis underscores translational repression of transcripts coding for ribosomal proteins, mitochondrial components, and cell cycle factors in older mice

**Figure 4:**
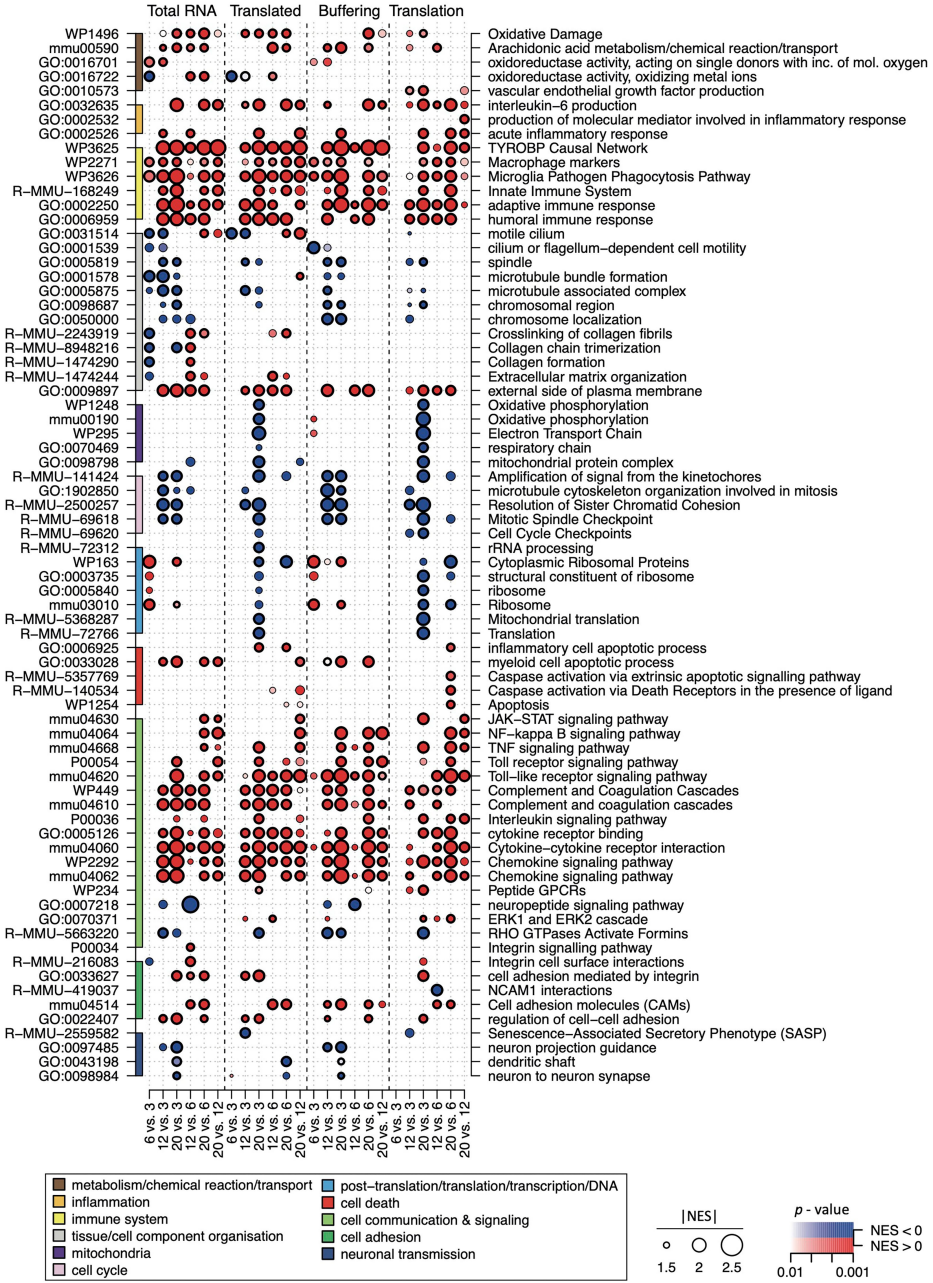
GSEA of the transcriptome (“Total RNA”), translatome (“Translated”) and the “Buffering” and “Translation” regulatory modes defined with anota2seq. 74 significantly enriched terms (left: ID numbers) across time comparisons (bottom horizontal axis) are shown. Normalized enrichment scores (|NES|) for each term are displayed and are proportional to the circle size. The colormap refers to *p*-values indicated at the bottom, circles with a black border line refer to terms with FDR < 0.05. Red: terms associated with up-regulated genes (+ NES score); blue: terms associated with down-regulated genes (− NES score). 74 terms were manually allocated to 11 functional classes (colors in left vertical axis) and described in the bottom right legend.

The identification of DEGs based on threshold fold changes and statistical significance may be well suited to uncover key factors/pathways and biomarkers, nevertheless biologically relevant coordinated responses in the gene expression landscape may be overlooked. We therefore performed a comprehensive GSEA analysis of the annota2seq data across all age comparisons, considering ranked lists of all 16,801 genes ordered by changes in (i) ‘total RNA’ levels (total RNA transcriptome, *x*-axis of scatter plot showed in Figure 3B; Supplementary Figure 4), (ii) polysomes, referred to as ‘translated’ RNA (*y*-axis of scatter plot in Figure 3B), (iii) net ‘translation’ that eases changes in the total RNA levels, and (iv) ‘buffering’ that alleviates translational response effects (Oertlin et al., 2019). Overall, 469 terms were selected with an FDR < 0.05 in at least one paired-age comparison, which were then manually allocated to 16 functional classes (Figure 4 depicts a selection of 74 themes in 11 functional classes; the complete data is shown in Supplementary Figure 6).

While many biological processes were distributed across all expression categories, such as the continuous decrease in the expression of genes related to microtubule bundle, cilium organisation, and the steadily increase in inflammatory and immune response genes, selective variation in total and translated mRNAs changes and the associated net effects (translation/buffering) were detected (Figure 4). Most prevalent was the translational repression of mRNAs coding for ribosomal proteins (RPs) in older animals (i.e., particularly prominent by comparing 20- vs. 3- or 6-month-old mice). Translational repression of RPs was previously recognised in other tissues and could thus frame a hallmark of aging (Anisimova et al., 2020; Woodward and Shirokikh, 2021). Interestingly though, we found that the observed translational repression is preceded by a significant increase of respective mRNA levels from young adults to middle-aged animals (i.e., comparing 6 vs. 3 months). Therefore, the age-induced translational repression counters the transcriptome changes in young adults, suggesting a turning-point in the gene expression programme in early to middle-aged animals. Likewise, we observed that mRNAs coding for mitochondrial components became translationally repressed after 12 months, but the respective RNA levels remained unchanged or even slightly increased from young toward middle-aged adults (6 vs. 3 months). Furthermore, cell-cycle checkpoint factors were also translationally repressed in older animals (12-months old), that could be linked to the previously observed reduced cell division rates with age (Tomasetti et al., 2019), while cell-death components and caspase activated processes became translationally activated with age (comparing 20- vs. 6-month animals). Overall, prominent diffraction and reprogramming of translation seems to occur between middle-age toward old animals including the propensity to deactivate translation of key cellular components required for cell growth, propagation, and maintenance, while factors related to cell degradation become activated with age.

### Alternative splicing events increase with age, and occur in sets of genes distinct from differentially expressed genes

**Figure 5:**
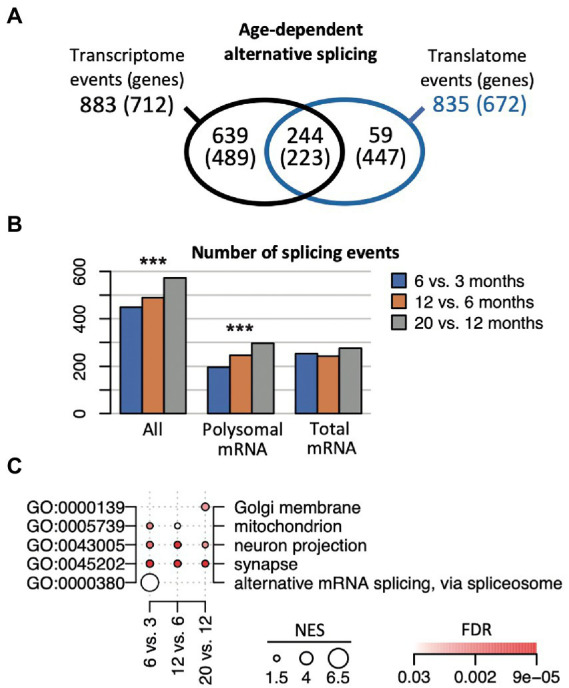
Age-dependent alternative splicing (AS) in the transcriptome and translatome. **(A)** Number of identified AS events and genes (in brackets) across the transcriptome and translatome (polysome). **(B)** Number of identified splicing events at the indicated time comparisons, considering all genes (all), the ones identified in the polysomes and transcriptome. ***p < 10^−3^ (Chi-square test). **(C)** Selection of enriched GO terms (FDR < 0.05) across indicated time comparisons considering all AS genes identified in the transcriptome and the translatome. NES for each term proportional to the circle size; FDR values are color coded.

Changes in splicing activity could substantially reshape gene expression during aging (Stilling et al., 2014). Nevertheless, how alternative splicing (AS) events are synchronized between the transcriptome and translatome during aging is not known. Therefore, we implemented the SUPPA2 pipeline to search for AS events (Trincado et al., 2018). This analysis across all the different age comparisons revealed 1,474 significant splicing events in 1,138 genes (*p* (BH) < 0.05), corresponding to 6.7% of all expressed genes (annotated list of all AS events displayed in the Supplementary Table 5). 883 events in 712 genes were identified comparing transcriptomes at different ages, 835 events in 672 genes were identified comparing the translatomes, while 244 events (16.5% of all events, corresponding to 223 genes) were observed in both the transcriptome and translatome, representing a significant overlap (Fisher’s exact test, *p* = 10^−256^ considering 65,311 possible events; Figure 5A). Importantly, the selected AS genes were substantially different from DEGs: only 66 (5.7%) and 33 (2.9%) of all AS genes were identified as DEG in age comparisons with anota2seq and edgeR, respectively. This finding is in line with a previous report on the hippocampal transcriptome of aged mice (Stilling et al., 2014), underscoring independent control of AS events and DEGs during aging. Interestingly though, we recorded a slight but significant increase in the overall number of AS events with age (increase by 49.6%; Chi-square test, *p* = 3.9 × 10^−4^) that was most prevalent comparing translatomes (increase by 53%; Chi-square test, *p* = 3 × 10^−5^), while only a minor increase was observed in the corresponding transcriptomes (1.5%; Chi-square test, *p* = 0.3; Figure 5B). This tendency toward enhanced alternative splicing in older mice describes henceforth a reverse trend to less prevalent changes in gene expression at later ages, possibly indicating a shift to splicing control in older animals.

We next sought whether functional themes were associated with time-dependent splicing events (Figure 5C; complete list of overrepresented GO terms is given in Supplementary Table 6). On the one hand, we identified functionally related themes among all AS genes that remained consistent over time and were detected in the transcriptome as well as the translatome. This category included gene transcripts associated with neurons (‘neuron projection/part’) and the ‘synapse’, underlining constant AS activity on genes with neuronal functions and in accordance with previous observations (Stilling et al., 2014). On the other hand, we observed functionally related groups among all AS genes that were prevalent at certain age-comparisons and preferentially detected in either the transcriptome or translatome. For instance, AS changes in early lifetimes were particularly allocated to ‘alternative mRNA splicing, *via* spliceosome’ (6 vs. 3 months) and mRNA binding (12 vs. 3 months), including serine/arginine-rich splicing factors/regulators (*Srsf1*, *Srsf6, Rsrp1*), ELAV-like family members (*Celf1*, *Celf2*), and splicing associated RBPs (e.g., *Rbm7, Rbmx*), and mainly driven by AS events seen in the transcriptome (Supplementary Table 6). Likewise, genes associated with the ‘mitochondrion’ were preferentially AS up to 12 months and code for mitochondrial ribosomal proteins (*Mrpl13*, *23*, *30*, 58), cytochrome c oxidase subunits (*Cox6b*2, *Cox7a2*), sirtuin 3 (*Sirt3*, a regulator of mitochondrial transcription), and peroxiredoxins (*Prdx1*, *Prdx6*), which are implicated in the removal of superoxide radicals. AS events at later-ages (e.g., 20 vs. 12 months) were enriched for genes allocated to the ‘Golgi membrane’ and included transcripts coding for syntaxins (*Stx16*, *Stx18*) and other neuronal related Golgi-proteins like N-acetylgalactosaminyltransferases (*Galnt6*, *Galnt13*); while AS events in the translatome at that time comparison were overrepresented for mitotic ‘spindle’ components (FDR = 0.0046). Thus, the data suggests that time-dependent AS activity on certain compartment-specific gene products could be associated with alterations of splicing activity in the aging hippocampi.

### Distinct splicing event types are associated with functionally related genes

**Figure 6:**
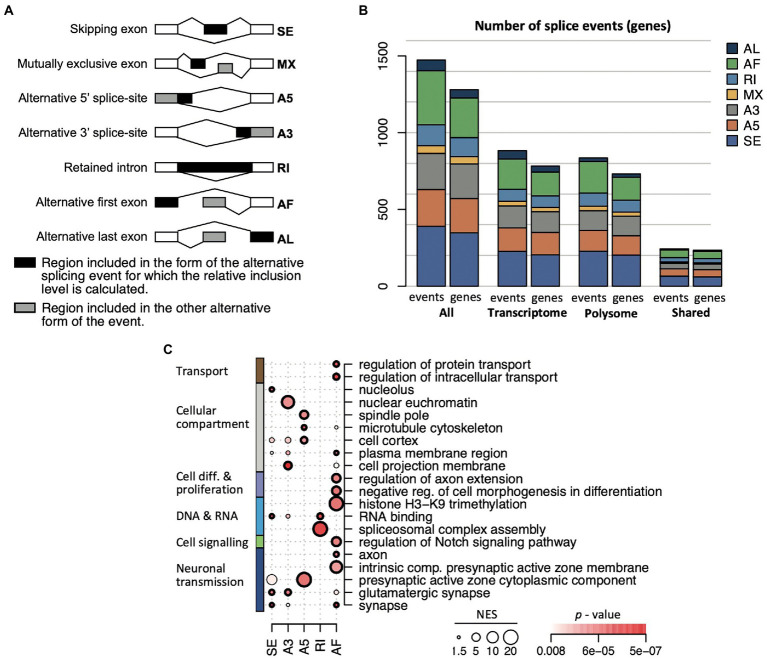
AS event types in the transcriptome and translatome. **(A)** Scheme of the seven different event types identified with the SUPPA2 analysis. The relative inclusion levels (Ψ) concern the region depicted in black; a gray area indicates alternative forms of the event. **(B)** Number of events and genes associated with each of the seven event types across the transcriptome and translatome (polysome). **(C)** Selection of 20 enriched themes overrepresented among genes displaying different splicing event types. The diameter of the circle is proportional to the NES score, the colormap refers to the *p*-value, circles in bold refer to FDR < 0.05.

We next wondered about differences among transcripts associated with the seven splicing event types informed from the SUPPA2 analysis (Figure 6A; selected examples are displayed in the Supplementary Figure 7). Overall, the fraction of transcripts allocated to different event types was comparable between the translatome and transcriptome; exon skipping (SE) being the most frequent and mutually exclusive exons (MX) being the least prevalent AS event during aging (Figure 6B). Most AS involved one splice event per gene (>90%, simple events) and only 99 genes were associated with at least two different splice events (i.e., 60 out of 712; 8.4% genes identified in the transcriptome; 51 (out of 672; 7.6%) in the translatome; and 12 genes in both). The latter included transcripts coding for proteins regulating gene expression/transcription/translation, apoptotic processes, cell aging and cellular senescence, as well as processes relevant to the amyloid deposition in aging and Alzheimer’s disease (e.g., *Flot2*, *Rab11a*, *Mdm2, Apbb1*).

Interestingly, substantial differences among functional themes were associated with the different AS event types (Figure 6C; a display of all 51 overrepresented themes is given in Supplementary Figure 8). The exon skipping (SE) events were overrepresented for ‘neuron’ and ‘synapse’ components (FDR < 0.05) and RBPs (38 genes, 11% of all 345 SE genes, FDR = 0.021) with splicing regulators (e.g., *Srsf1*, *Hnrnpr*, *Imp4*, *Mbnl2*, *Matr3*, *Puf60*, *Lsm4*) and protein synthesis components (e.g., *Rpl14*, *Eif4g1*, *Eif4enif1*) including DEAH-box translational regulators (*Dhx32*, *Dhx37*). Transcripts coding for RBPs were also enriched among intron retention (RI) splicing events, covering 19 RBPs (20% of all annotated genes in RI; FDR = 0.0084), five of them acting in spliceosome assembly (*Celf1*, *Celf2*, *Luc7l3*, *Rbm5*, *Srsf6*; FDR = 0.05; Figure 6C). Alternative first exon selection (AF) was the second most observed splicing event, and the associated genes commonly coded for proteins involved in regulation of intracellular transport, peptide/protein transport and secretion as well as enzyme and kinase regulator activity; and they were confined to specific cellular compartments including the dendritic tree, axon, somatodendritic compartment, presynaptic active zone, and glutamatergic synapse (FDR < 0.05; Figure 6C). Notably, AF events were registered in four transcripts coding for histone H3-K9 methylation components (*Kdm4a*, *Kdm4c*, *Mecp2*, *Suv39h2*), corroborating a potential role in chromatin remodeling during aging (Keenan et al., 2020); seven transcripts associated with the regulation of the Notch signaling pathway (e.g., *Dkl2*, *Hes1*) which plays an important role in aging (Balistreri et al., 2016); and seven transcripts encoding ribosomal proteins, whose expression is translationally repressed in aged animals (cytoplasmic *Rpl5*, *Rpl7*, *Rpl18a*, *Rpl37rt*; and mitochondrial *Mrpl23*, *Mrpl30*, and *Mrps5*). Alternative 5′ splice-site (A5) events included mRNAs coding for proteins acting at the cell cortex, cytoskeleton, and the presynaptic active zone cytoplasmic component, with further specification in the translatome for microtubular components such as the spindle and spindle pole body (FDR < 0.05 in the translatome); whereas alternative 3′ splice-site selection (A3) was particularly seen among nuclear euchromatin components (4 genes: *Nsmf*, *Prdx*, *Rbmx*, *Rbmxl1*), cell projection and leading-edge membrane or organelle outer membrane (Figure 6C). The lowest number of splicing events were allocated to alternative last exon (AL) and mutually exclusive exons (MX) event types. Here, no significant functional themes among associated transcripts could be retrieved with FDR < 0.05. However, AL associated genes showed a tendency for enrichment of proteins acting in transport vesicles (*p* < 0.0011, FDR = 0.39), while MX events referred two splicing events in the *Apbb1* transcript, which is known to be AS during aging (Stilling et al., 2014), as well as in the ionotropic glutamate receptor complex (*Dlg3*, *Eps8*, *Porcn*; *p* = 0.00038, FDR = 0.22). Overall, the different splicing event types were allocated to distinct functionally related genes, many of which bear biological relevance in aging in the brain and hippocampus. In most cases, those functional themes were commonly identified among AS genes in the transcriptome and translatome – suggesting functional coordination – although the actual transcripts undergoing significant AS can be different.

### RNA targets of the RNA-binding proteins CIRBP and RBM3 represent a significant portion of identified genes with AS events during aging

As our differential expression analysis suggested dynamic expression of RBPs, possibly leading to variation of post-transcriptional control during aging, we wondered whether the RNA targets for RBPs may be associated with changes in expression or splicing. Focusing on the cluster of RBPs showing higher transcript abundance in 12 month-old mice followed by a drastic decline at 20 months (Figure 2, Cluster 4), the experimentally determined mRNA targets for RBM3 and CIRBP from fibroblasts (Morf et al., 2012; Liu et al., 2013) were available and retrieved *via* POSTAR3 (Zhao et al., 2022). The mRNA targets for those two stress-related proteins were underrepresented among DEGs during aging (i.e., 44 RBM3 mRNA targets, 121 CIRBP mRNA targets); but they overlapped significantly with the list of AS genes targeted either by RBM3 (229 genes, 20.2% of all AS genes associated with 310 events; *p* = 2.3 × 10^−8^, hypergeometric distribution), or CIRBP (369 genes, 32.4%; related to 490 events; *p* = 7 × 10^−11^, hypergeometric distribution; RNA targets marked in Supplementary Table 5). Among them, 140 gene transcripts were targets for both RBPs (12.6%) and preferentially code for RBPs (FDR < 10^−6^) including splicing factors (FDR < 10^−5^) and/or are part of synapses (FDR < 2 × 10^−4^). These significant correlations suggest critical roles for these stress regulated RBPs in splicing regulation of neuron-related transcripts during aging and hence comprise valuable targets for further evaluation.

### No substantial differences in poly(A) site usage during aging

Differences in polyadenylation site selection during aging was previously reported in the brain cortex, claiming a significant shift toward distal UTR usage at 52 weeks (i.e., 12 months) compared to 6-week-old mice (Shafik et al., 2021). Furthermore, it was suggested that RBM3 and CIRBP could regulate circadian gene expression by controlling alternative polyadenylation (APA) in mouse embryonic fibroblasts (Liu et al., 2013). Thus, we wondered whether differences in 3′UTR end selection may apply during age progression, and we searched for differences in poly (A) site selection using LABRAT, a pipeline that quantifies the usage of APA and cleavage sites in RNA-seq data (Goering et al., 2021). Essentially, we did not observe a global shift in APA selection between different age groups (Supplementary Figure 9). Only ten transcripts significantly changed polyadenylation sites across age comparisons (*p* (BH) < 0.05; Supplementary Table 7). Specifically, transcriptome analysis suggested APA in *Fam76b* (20 vs. 12/6 months), *Nmt2* (20 vs. 3 months), *Mindy2* (12 vs. 3 months), and *Gtf3c2* (6 vs. 3 months); the latter being a mRNA target for RBM3, while the other transcripts are known CIRBP targets. Translatome analysis suggested significant changes in UTRs for *Lnx1* and *Dcp1* (12 vs. 3 months), *Gemin8* (12 vs. 6 months), *Tmem201*, *Comtd1*, and *Taf6l* (all 20 vs. 6 months), the latter being a RBM3 target. In conclusion, these results suggest only minor changes of poly (A) site selection between 3 and 20 months, suggesting rather decisive poly (A) site selection in young adults for an extended period of life.

## Discussion

Our comprehensive analyses of RNA sequencing data in adult female mouse hippocampus across four ages showed a substantial concordance in relative changes of transcript levels of the transcriptome and translatome, while divergences imposed by age-dependent changes in translational efficiencies concerned genes encoding ribosomal and mitochondrial components, cell-cycle and apoptosis related factors, as well as a subset of lncRNAs. Furthermore, a divergence of the effects of aging was uncovered for the alternative splicing in the translatome and transcriptome, and transcripts targeted by the RNA-binding proteins CIRBP and RMB3 represented a significant subset of genes displaying splicing events with aging. Finally, aging was only marginally associated with changes in alternative polyadenylation sites. Thus, the herein outlined combination of various RNA sequencing analyses highlight the complex picture for multi-level post-transcriptional control of hippocampal genes during chronological aging, and it provides a unique resource for further investigation of affected pathways and its associations with age-associated diseases.

In accordance with previous reports considering the transcriptome (Stilling et al., 2014), only a relatively small fraction of genes was differentially expressed from young to aged adults in the mice female hippocampi. Most genes were commonly upregulated with remarkable parallel and progressive changes in the transcriptome and translatome and code for proteins contributing to immune system processes and neuroinflammation (Toll-like receptor signaling pathway, leukocyte activation, cytokine production); while the few downregulated genes were associated with neurogenesis and consistent with age-related changes observed at the transcriptome level in one of the main neurogenic niches (i.e., subgranular zone of the hippocampal dentate gyrus) in humans (Bitar et al., 2022). Overall, these findings are consistent with aging being the major risk factor for neurodegeneration/dementia and that neuroinflammation plays a crucial role in the development Alzheimer’s disease and frontotemporal dementia (Leyns and Holtzman, 2017; Leng and Edison, 2021).

Our analysis also revealed age-dependent expression of more than 200 lncRNAs and pseudogenes. This includes *Neat1* that plays a key role in the modulation of neuronal activity and hippocampal-dependent memory formation in mice (Barry et al., 2017; Butler et al., 2019) and has been implicated in Alzheimer’s disease (Riva et al., 2016; Yang et al., 2017; Wu et al., 2019). Age-associated changes in lncRNAs expression were previously reported in mouse and human tissues and could – as previously reported – contribute to immune function *via* modulation of the NF-kappaB signaling pathway, as well as inflammation and transcription (Hammond et al., 2020; Marttila et al., 2020; Zhou et al., 2020; Cai and Han, 2021). Besides potential diverse regulatory functions of lncRNAs in translation, short ORFs translated from lncRNA could give rise to micropeptides (referred to as proteins <100 amino acids; Minati et al., 2021). Hundreds of different micropeptides produced from lncRNAs or upstream ORFs have very recently been found to be expressed in human brain cortex. Many of them contain RGG-rich peptides, which comprises a potential RNA-interaction domain that may infer RNA regulatory functions (Duffy et al., 2022). It remains to be determined whether the herein identified polysomal-associated lncRNAs give raise to micropeptides or bear other regulatory functions related to the aging phenotype.

The analysis of translational efficiencies using the anota2seq algorithm refined detection of convergent and divergent alterations in the transcriptome and translatome associated with age. Besides various convergent responses including well-known transcriptional inferred responses (e.g., immune and inflammatory response) that mainly changed in a mono-typic fashion (i.e., steadily increasing expression with age), subsets of DEGs displayed rather unique peak- or V-shaped expression trajectories. These non-monotonic age-related changes were observed in all three regulatory modes though were more prominent in the ‘translation’ and ‘buffering’ regulatory mode likely associated with post-transcriptional regulation. For example, an overall decline between 3 and 6 months was observed for DEG in the ‘translation’ and ‘buffering’ regulatory modes (where translation deviates from changes in the transcriptome) and preferentially coding for proteins located at the cilium and axoneme cellular compartments (C7, Figures 3C-E), which could go along with known changes in neural homeostasis and hippocampal neurogenesis (Kirschen and Xiong, 2017). In the ‘abundance’ regulatory mode, a notable example V-shaped gene expression profile concerned transmembrane and ion transporters, such as solute carriers (*Slc*) belonging to the largest family of transmembrane transporters that modulate essential physiological functions including nutrient uptake and ion transport (C1). The highly temporally controlled and coordinated expression of these transporters across chronological age further reflects the importance of age-related alterations in energy metabolism (Hu et al., 2020).

Discordant translational repression was also observed for specific genes involved in the modulation of memory and may contribute to neurodegenerative diseases. For instance, genes encoding proteins acting in calcium mediated signaling, which is consistent with disruption of age-related changes in long-term potentiation and deficits in hippocampal-dependent behavioral tasks in the aging mice (Pereda et al., 2019); and ribonucleoprotein (RNP) granules, that have been involved in the development of Alzheimer’s disease and frontotemporal dementia (Wolozin and Ivanov, 2019; Desai and Bandopadhyay, 2020). More ‘broad’ translational repression concerns mRNAs coding for ribosomal proteins (RPs) that was particularly prominent comparing 20- vs. 3- or 6-month-old mice. The translational repression of RPs has been previously observed in aged liver and kidney mice tissues and likely reflect changes in mTOR signaling as these transcripts contain 5′ terminal oligopyrimidine tract (5’TOP) motifs (Anisimova et al., 2020). Remarkably though, we further observed that this repression was preceded by elevated levels of respective mRNAs for up to 6 months, which could suggest that neurons of younger adults’ build-up a ‘reservoir’ of those mRNAs enabling rapid engagement and synthesis of RPs on demand. Since active translation is linked to memory formation, it is tempting to speculate that such ‘readiness’ is beneficial for learning, which is better performing in young adults. Conversely, translational repression of RPs at advanced age may promote alterations in synaptic plasticity and synaptic transmission in the hippocampus that could eventually lead to cognitive deficits (Schimanski and Barnes, 2010).

Alternative splicing is increasingly recognised as a key post-transcriptional regulatory element involved in aging, and its dysregulation has been identified as a key mechanistic feature in Alzheimer’s disease (Raj et al., 2018). Reminiscent to previous reports obtained with mouse skin, skeletal muscle and bone (Rodriguez et al., 2016), we observed a significant increase in the number of AS events with age, preferentially in the translatome. Notably, a recent study reported an age-dependent increase in the use of distal 3′ splice sites in mRNA targets in the nematode *C. elegans* (Ham et al., 2022). Maybe related to this finding or by coincidence, the highest number of distal splicing sites (A3) was identified at later ages, comparing 12 vs. 20-months old animals (50 and 51 events in the transcriptome and translatome, respectively). Further investigation would be required to consolidate a potential bias toward distal splicing events in the hippocampus of aged mice.

Remarkably, although AS genes significantly overlap, many different mRNA isoforms were found in the transcriptome and the translatome over the ages and across the seven different splice event types. While the underlying reasons for divergence of mRNA splicing isoforms have not been further explored, it may account for selection of AS transcript’s for association with ribosomes, which could be particularly selective in neurons (Floor and Doudna, 2016; Weatheritt et al., 2016). Whatsoever, despite the observed divergence of mRNA isoforms, in many cases AS genes commonly coded for functionally related classes of proteins with confounding functions in axons, dendrites and at synapses. AS could also generate numerous RBP isoforms, some of them acting in splicing, which suggests evolution of complex regulatory feedback networks. Moreover, the set of differentially spliced mRNAs were distinct from the differentially expressed ones, suggesting broad decoupling of those processes. In this regard, while we observed a striking expression profile for some RBPs, such as CIRBP and RBM3 (i.e., progressive increase up to 12-month-old followed by a sharp decrease at 20 months), their RNA targets represented a significant portion of genes displaying splicing events. Moreover, these potentially AS RNA targets were enriched for genes coding for RBPs. Along previous observations showing that RBM3/CIRPB could interact with intronic sequences in the nucleus and RBM3 is associated with the spliceosome (Zhu et al., 2016), it is possible that those RBPs could be part of a highly controlled RNA-splicing network controlling neuronal homeostasis and aging. The complex splicing-network could also create resilience to balance dysfunction imposed by diminished mitochondrial function and energy status of cells with age (Ferrucci et al., 2022).

Mitochondrial dysfunction plays an important role in aging and is anticipated to contribute to age-related neurodegenerative diseases (Cenini and Voos, 2019; Haas, 2019). Interestingly, we observed that nuclear genes coding for mitochondrial proteins were preferentially AS up to 12 months (Figure 5). Furthermore, and as previously seen for cytoplasmic RPs, we recorded substantial translational repression of gene sets related to mitochondrial physiology (i.e., genes coding for mitochondrial translation factors, the electron transport chain and oxidative phosphorylation) in 20-month compared to 3-month-old mice (Figure 4). While the decline in mitochondrial function during aging is well-documented (Frenk and Houseley, 2018), the potential association with splicing and translational control has not yet been established in other tissues and may therefore be critical in hippocampi and possibly other neuronal tissues. Maybe – as we speculate – the observed effects could also relate to alterations of mTOR signaling through translation initiation factor 4E-binding proteins (4E-BP) dependent translational regulation as likely inferred for translational repression of cytoplasmic RPs. Although the proposed relations need to be further investigated, it has been shown that TORC1 is linked to the expression of mitochondrial proteins and possibly splicing in cancer cells (de la Cruz Lopez et al., 2019).

In conclusion, our study highlights the complexity and importance of translational control and splicing in adult brains as compared to other post-transcriptional control points, such as polyadenylation site selection at the 3’end mRNAs to generate different 3’UTR. The definition of age-dependent gene clusters for translation and splicing regulation enables detailed studies on functional impact in hippocampi and its associated functions in memory formation and neurogenerative diseases. Nevertheless, our study was carried out only with one sex (female mice currently underrepresented in mice studies) and a relatively low sample size. To deepen our understanding of age-related alterations, future studies should consider a greater number of animals from both sexes, enabling the investigation of sex differences in aging. Furthermore, our study was focused on one brain tissue (hippocampus) and did not consider alterations of specific cell types in that tissue. An expansion of the study to other brain tissues may be feasible though currently limited by the good amounts of tissue/cells required for translatome analysis. Thus, the application and further development of single-cell ‘-omics’ approaches for transcriptome and translatome analysis could open up a new area to further our understanding of gene expression regulation in aging brain cells and associated diseases.

## Materials and methods

### Subjects

Four age groups of female C57BL/6 J mice (Charles River Laboratories, Margate, United Kingdom) were included in this study (*n* = 6, 84–91 days; *n* = 6, 23 weeks; *n* = 4, 59 weeks; *n* = 6, 88 weeks). Following arrival in the facility, mice were group-housed by age group for an 18-day habituation period, under a 12 h:12 h light/dark cycle, controlled ambient temperature (20–22°C) and humidity (55% ± 10%) in IVC ventilated cages (Optimice^®^ System, AnimalCare|Systems, Centennial, Colorado, United States). Cages were enriched with nesting material, red domed house, forage mix and aspen chew blocks. Food (A04 maintenance diet) and water were available *ad libitum*. Tissue collection was randomised by age groups and started at Zeitgeber (ZT) ZT1 (i.e., start of the collection 1 h after light onset) and was completed within 40-min to control for circadian rhythms. The hippocampus (as well as hypothalamus, cortex, liver, heart kidney, and surrenal glands) were collected in this order and immediately snap-frozen in liquid nitrogen and stored at −80°C. The experimental procedures were approved by the Animal Welfare and Ethical Review Body of the University of Surrey and were conducted in accordance with the UK Animals (Scientific Procedures) Act 1986.

### Extract preparation

Each hippocampus (mean ± SD: 41.1 ± 4.6 mg) was ground to a fine powder with a pestle in a mortar filled with liquid nitrogen and transferred to ice-cold lysis buffer (20 mM Tris–HCl (pH 7.5), 150 mM NaCl, 10 mM MgCl_2_, 0.1 mg/ml cycloheximide, 0.2 mg/ml heparin, 0.5% Triton-X-100, 0.1% sodium deoxycholate, 0.5 mM DTT, 100 U/mL RNasin (Promega #N2615), 1× complete protease inhibitor (Roche #11836170001), 10 U/mL DNase I (Promega #M6101)). Samples were thawed on ice. The suspension was homogenized and centrifuged at 12,500*g* for 5 min at 4°C. The supernatant was adjusted with lysis buffer to 1 ml. One fourth of the obtained samples was used for total RNA extraction with the Zymo RNA MiniPrep Isolation Kit (Zymo #R1) applying on-column DNase I digest. RNA was precipitated with 2.5 M LiCl at −20°C to remove residual heparin (see below).

### Polysomal profiling and RNA isolation

Polysomal profiling was performed as previously described (King et al., 2017). 0.75 mL of hippocampal extracts were layered on top of a linear 15–50% sucrose gradient prepared in 20 mM Tris–HCl, pH 7.5, 150 mM NaCl, 10 mM MgCl_2_, 0.1 mg/mL cycloheximide and 0.2 mg/mL heparin. The samples were centrifuged at 100,000*g* for 2.5 h at 4°C in a SW41 swinging bucket rotor, and 0.8 ml fractions of the gradient were collected while continuously recording the absorbance at 254 nm (A_254_) with a flow cell UV detector (ISCO). For subsequent RNA sequencing of polysomes, 0.4 ml (50%) from each of the fractions 7 to 12 representing polysomes were combined and RNA was isolated by addition of 3 M guanidine hydrochloride, 50% ethanol, 15 μg glycoblue (Ambion), vortexed and incubated overnight at −20°C. The RNA was pelleted by centrifugation at 16,000*g* for 90 min at 4°C, resuspended in RNase-free water (Sigma) and subjected to a second precipitation with 2.5 M LiCl at −20°C overnight to remove residual heparin. After centrifugation, the RNA pellet was washed with 75% ethanol, dried and resuspended in RNase-free water and treated with DNase (TURBO DNA-free, Ambion #1907). RNA was quantified with a Quantus (Promega) device and quality assessed with a Bioanalyzer for calculation of RNA integrity numbers (RIN). Data attrition occurred for 2 samples: one sample for the 20 month-old group was lost during preparation; one sample for 3-month-old group had low-quality polysomal profile (polysomal profiles are given in Supplementary Figure 1).

### Monitoring gradients with RT-PCR

RNA was isolated from individual polysomal fractions as described for pooled fractions and resuspended in 20 μL RNase-free water and DNase-treated. 9 μL of the isolated RNA was reverse transcribed (RT) using a mixture of oligo(dT)_18_ and random hexamer primers with the Precision nanoScript 2 Kit (Primer Design) for 2 h at 42°C. PCR was then performed with gene-specific primers for *Rpph1* (forward: 5′-GAGGGAAGCTCATCAGTGGG-3′, reverse: 5′-GCCCTAGTCTCAGACCTTCC-3′); *Eef2* (forward: 5′-GGTACTTTGACCCAGCCAACG-3′; reverse: 5′-AAGATGGGGTCCAGGATCAGC-3′), and *28S* rRNA (forward: 5′-CAAAGCGGGTGGTAAACTCC-3′; reverse: 5′-CTCTTAACGGTTTCACGCCC-3′) with GoTaq Green (Promega, M7822). The following temperature program was used: 2 min at 95°C, followed by 30–35 cycles of the sequence 95°C for 30 s, 59°C for 30, and 72°C for 30 s and a final extension for 5 min at 72°C in an Applied Biosystems Veriti Thermocycler.

### RNA sequencing, alignment, and mapping

Total RNA samples (*n* = 20; representing the transcriptome) and RNA from matched polysomes (i.e., translatome; *n* = 20, Supplementary Figure 1) were subjected to RNA sequencing (Wellcome Trust Sequencing Facility, Hinxton, Cambridge). RNA-seq was thus performed on matched samples from 20 subjects, comprising five samples from mice at the age of 3 months, six mice at 6 months, four mice at 12 months, and five mice at 20 months. Poly(A) RNA was selected from total RNA, converted to cDNA and amplified for library preparation. Libraries were sequenced on an Illumina HiSeq 4000 platform using 75 nt paired-end sequencing runs. Quality checks were performed *via* FastQC (v 0.11.4; Andrews, 2010). Reads were mapped to the mouse genome (Gencode release M12 (GRCm38.p5) primary assembly genome and comprehensive gene annotation) using STAR [v 2.5.2b] (Dobin et al., 2013), generating output of genomic alignments in genome and transcriptome coordinates. The function featureCounts from the R package Rsubread [v 1.16.1] (Liao et al., 2014) was used to assign mapped sequencing reads to genome features. Genome features were defined by the tool’s in-built gene annotations for the mouse genome (NCBI RefSeq gene annotations Build 38.1) resulting in the mapping of reads to 27,179 genes. Genomic features were annotated using the R package org.Mm.eg.db [v 3.10.0] and GenBank (accessed 20-25th November 2019 *via* the R package Annotate [v 1.64.0]). Filtering of low abundant genes was performed by keeping genes with at least $N=\underset{1\leq i\leq 20}{\mathrm{median}\;}\left(8\times 10^{6}/{\mathrm{total counts}}_{i}\right)$ counts per million (CPM) in at least 4 samples derived from total (i.e., transcriptome) or polysomal RNA (i.e., translatome). A total of 16,899 genes remained after filtering. Data were normalized using the trimmed mean of *M*-values (TMM) normalization (Robinson and Oshlack, 2010).

### Batch-effects and data attrition

Principal component analysis (PCA) and Pearson correlation of TMM normalized log_2_ CPM were used to evaluate batch effects, outliers, and sample similarity. PCA analysis showed a clear separation of the transcriptome and translatome samples. However, it also revealed a batch effect of gradient date and containing two outlier samples (Supplementary Figure 2). In this regard, samples were classified as outliers if their average pairwise correlation to all samples within their set (i.e., total mRNA or polysomal mRNA samples) fell more than 1.5 times the interquartile range below the first quartile of the average pairwise correlation of the set. The two outlier samples (*n* = 1 subject aged 20 months and *n* = 1 at 6 months) were associated with lower quality of the gradient for polysome preparation and excluded from further analysis (Supplementary Figure 1). Batch effects were subsequently corrected using the ComBat method from the R package sva [v 3.34.0]. We used ComBat as it uses an empirical Bayes approach to avoid over-correction with small batches. Data were then pre-processed again, leading to a total of 16,801 genes (Entrez IDs) across remaining 18 matched samples for further analysis. PCA analyses showed that the batch correction was successful, and samples have higher similarity within age groups with very similar distribution within total mRNA and within polysomal samples (Figure 1C; Supplementary Figure 2). For differential expression analysis between age groups performed with edgeR, batch effects were hence incorporated in the model. For anota2seq analysis, batch effects were removed prior to analysis using Combat function from the R package sva [v 3.34.0] (Jeffrey T Leek et al., 2012). Batch correction for the total mRNA and polysomal mRNA samples was performed independently.

### Differential expression analysis between age groups with edgeR

Differential expression was performed using the edgeR quasi-likelihood pipeline (Robinson et al., 2010; Chen et al., 2016). Briefly, a quasi-likelihood (QL) negative binomial generalized log-linear model was fit to the count data using the edgeR Bioconductor package [v 3.28.0]. Gene-wise Empirical Bayes quasi-likelihood F-tests were then performed to specific contrasts (20 vs. 3 months, 12 vs. 3 months, 6 vs. 3 months, 20 vs. 6 months, 12 vs. age 6 months, and 20 vs. 12 months). The QL dispersion estimation and hypothesis testing were performed with the functions glmQLFit and glmQLFTest. The batch effects were controlled by adding them to the design matrix, such that differential expression is tested while adjusting for differences between batches. Genes classified as having a significant change in expression with age were defined as those having a Benjamini-Hochberg (BH) corrected *p* < 0.05 and abs(FC) > log_2_(1.2).

### Anota2seq analysis

Anota2seq analysis was performed using the anota2seqRun function [anota2seq v 3.33.1] (Oertlin et al., 2019). The input consisted of the batch corrected, filtered TMM normalized log_2_ CPMs (16,801 genes), hence, the filtering and normalization within the anota2seq pipeline were switched-off. Significant events were selected based on all default settings except for a decrease in the BH adjusted *p*-value threshold (maxPAdj = 0.05, default 0.15) and an increase in the minimum effect for inclusion (minEff = log_2_(1.2), default 0). Model assumptions were assessed using the QC plots produced by the tool.

### Aging trajectories and clustering

For each gene, a temporal expression profile was generated by averaging the TMM normalized log_2_ CPM values of replicates within each age group and z-scoring the resulting four averages. Total mRNA temporal profiles were used as input for the clustering analysis of genes significant in anota2seq abundance mode, anota2seq buffering mode, and edgeR, while polysomal mRNA was used for the clustering analysis of genes significant in anota2seq translation mode. Temporal profile matrices from both total mRNA and polysomal mRNA samples were used in the visualization of the results *via* heatmaps and scatterplots. Clustering based on circular Self-Organizing Maps (SOM) was performed on the temporal profiles (Möller-Levet and Yin, 2005). Briefly, the circular SOM based on the co-expression coefficient was used for grouping and ordering expression profiles based on their temporal properties. Linear interpolation was applied for profile modeling in the calculation of the co-expression coefficient. For each gene list, the number of clusters was established using the Bayesian Index Criterion (BIC; Schwarz, 1978).

### Detection of alternative splicing events

RSEM [v 1.2.19] (Li and Dewey, 2011) was used to quantify transcript expression from the STAR alignments. Given the abundances for all transcript isoforms, SUPPA2 [v 2.3] (Trincado et al., 2018) was used to test differential relative inclusion values of alternative splicing events across age groups. Comprehensive gene annotation on the mouse primary assembly (Gencode release M12 (GRCm38.5)) was used to generate the different splicing event types. Relative abundances of the splicing events are described in terms of a percentage or proportion spliced-in index (PSI). PSI per event is calculated as the ratio of the transcript abundances from one form of the event to the combined transcript abundances of both forms of the event. Differential splicing is given in terms of the difference of these relative abundances, or ΔPSI, between conditions.

### Detection of alternative polyadenylation sites

LABRAT was used to quantify the usage of alternative polyadenylation (APA) sites and identify genes whose usage of these sites varies across experimental conditions (Goering et al., 2021). APA sites for each gene are defined using transcript-terminal-fragments. These fragments correspond to the final two exons of every transcript. The expression of these transcript-terminal-fragments is quantified using Salmon (Patro et al., 2017). Close terminal-fragments (with 3′ ends within 25 nt of each other) are grouped together to define a single APA site. The term *psi* is used to quantify a gene’s relative APA site usage. Genes that show exclusive usage of the most gene-proximal APA site are assigned a *psi* value of 0 and those that show exclusive usage of the more distal-gene APA site are assigned a *psi* value of 1. Depending on the relative usage of both sites, *psi* can take a value between 0 and 1. LABRAT was further applied to compare psi values of experimental replicates across experimental conditions to identify genes that show statistically significant (*p*(BH) < 0.05) different *psi* values.

### Gene set overrepresentation and functional enrichment analysis

Gene sets were searched for overrepresented GO, KEGG pathways and transcription factor binding sites with Webgestalt using Entrez IDs as identifier and an FDR < 0.05 as default cut-off (Liao et al., 2019). Annotations obtained for the 16,801 mouse genes (Entrez IDs) from filtered and batch-corrected RNA-seq data were used as the background gene list. *p*-values were determined with hypergeometric test and FDRs represent BH corrected *p*-values for multiple-testing.

GSEA was performed with the ranked list of genes for each of the four regulatory modes (total RNA, translated, buffering, translation) across all time comparisons, considering GO and pathway annotations (Wikipathways, KEGG, Panther, Reactome) implemented in Webgestalt (Liao et al., 2019). Data were initially retrieved with FDR < 0.15 across all conditions. All terms with FDR < 0.05 in any condition were selected, and NES, *p*-values and FDRs were retrieved across all data with R to generate a GSEA matrix. Terms were classified manually into 16 categories (Supplementary Figure 6), representatives for 11 categories are shown in Figure 4.

## Data availability statement

The data presented in this study can be found in the European Nucleotide Archive (ENA) at EMBL-EBI under accession number PRJEB54003 (https://www.ebi.ac.uk/ ena/browser/view/PRJEB54003). Processed data presented in this study are included in the Supplementary material/Figures.

## Ethics statement

The animal study was reviewed and approved by Animal Welfare and Ethical Review Body of the University of Surrey in accordance with the UK Animals (Scientific Procedures) Act 1986.

## Author contributions

AG and RW-S designed and supervised the study. RW-S performed mice work. HK and VI optimized and run polysome profiles and performed RNA isolation. CM-L performed bioinformatic analysis of RNAseq data. AG and CM-L performed statistical analysis of the data. AG and RW-S provided a biological interpretation of the data. AG, CM-L, HK, and RW-S wrote sections of the manuscript. AG and CM-L revised the manuscript. All authors contributed to the article and approved the submitted version.

## Funding

The work was funded by the Leverhulme Trust to AG, RW-S (RPG-2014-267); and a Royal Society Wolfson Research Merit Award to AG (WM170036).

## Conflict of interest

The authors declare that the research was conducted in the absence of any commercial or financial relationships that could be construed as a potential conflict of interest.

## Publisher’s note

All claims expressed in this article are solely those of the authors and do not necessarily represent those of their affiliated organizations, or those of the publisher, the editors and the reviewers. Any product that may be evaluated in this article, or claim that may be made by its manufacturer, is not guaranteed or endorsed by the publisher.
