# Supplementary material for: A post-transcriptional regulatory landscape of aging in the female mouse hippocampus
Source: Front Aging Neurosci. 2023 Mar 24;15:1119873. doi: 10.3389/fnagi.2023.1119873 (PMC10135431; doi:10.3389/fnagi.2023.1119873)
Supplement: Supplementary file 8 [file Data_Sheet_1.docx]

Description of Supplementary Material

**A post-transcriptional regulatory landscape of aging in the female mouse hippocampus**

Raphaelle Winsky-Sommerer, Helen A. King, Valentina Iadevaia, Carla Möller-Levet, André P. Gerber*

*** Correspondence:** a.gerber@surrey.ac.uk

# Supplementary Figures (data sheet 2; pdf)

**Supplementary Figure 1.** Polysomal profiles of female mice hippocampi at indicated ages. Letters on the right hand-side correspond to the process sample number referring to RNA-seq samples; the letter code relates to sample labels for PCA analysis (Supplementary Figure 2). Samples with a star (*) were removed after batch-correction. Fraction numbers are indicated at the bottom; fractions 7-12 were collected for RNA-seq representing the translatome.

**Supplementary Figure 2.** Batch correction and outlier analysis of RNAseq samples based on PCA.

**Supplementary Figure 3.** Distribution of *p*-values from all age pairwise contrasts in total mRNA and in polysomal mRNAs. Differential expression was evaluated with the edgeR quasi-likelihood pipeline for 16,801 expressed genes.

**Supplementary Figure 4.** Relative changes of transcript levels in the transcriptome (total RNA) and translatome (polysomal RNA) in paired-age comparisons identified with anota2seq. Genes allocated to the indicated regulatory modes are depicted with different colors. The number of genes in each mode is indicated within brackets.

**Supplementary Figure 5.** Clusters of gene expression profiles identified with anota2seq. Heatmap and averaged temporal profiles of genes identified in **(A)** ‘abundance’ mode (929 genes) and **(B)** ‘buffering’ mode (601 genes) during aging. Clustering was based on total RNA (transcriptome) temporal profiles (left), and corresponding translatome data are shown to the right. The average profiles for each cluster are shown to the left with a continuous line for total RNA, a dashed line for polysomal RNA and error bars showing 95% CI. To the right, significantly enriched GO terms in each cluster. GO categories: BP, biological process, CC, cellular compartment, MF, molecular function. In black, *p* < 0.01 and FDR < 0.05; in grey, *p* < 0.01 but FDR > 0.05.

**Supplementary Figure 6.** Complete GSEA matrix comprising 469 enriched terms manually allocated to 16 functional classes.

**Supplementary Figure 7.** Examples of AS events identified with SUPPA2. Bar plots show the expression level of the indicated gene transcripts (colored bars with ENSEMBL accession numbers) across all replicate RNA-seq samples for the indicated age groups. The AS event type and *p*-value for the test of differential relative inclusion values across age groups are displayed below the gene symbol. Transcriptome (total RNA) events are shown to the left; translatome (polysomal mRNA) events to the right.

**Supplementary Figure 8.** Display of all 51 themes overrepresented across the seven splicing event types (extension of Figure 6C). Functional themes were identified among all genes of the indicated splicing category across all age comparisons and selected with *p* < 0.01, FDR < 0.05. The diameter of the circle is proportional to the |NES| score, the colormap refers to the *p*-value, circles in bold refer to selected FDR < 0.05.

**Supplementary Figure 9.** Alternative polyadenylation (APA) site usage across four ages in the hippocampus. Cumulative frequency plot representing the cumulative fraction of genes (*y*-axis) with averaged psi values (*x*-axis) at the four different ages (colored lines). Transcriptome (left, n = 4,912) and translatome (right, n = 4,714). The psi value denotes the fraction of distal *vs*. proximal APA site usage; psi = 0 indicates exclusive usage of proximal APA, psi = 1 of distal APA site.

# Supplementary Tables

**Supplementary Table 1.** EdgeR analysis data file and raw data (cpm). The worksheet ‘EdgeR_data’ shows differential gene expression analysis results for 16,801 filtered genes for the transcriptome and translatome (polysome). Information includes gene Entrez ID, Gene symbol, Ensembl ID number, gene biotype and log_2_ fold-changes for paired age comparison with *p*-values, and BH corrected *p*-values. A score of ‘1’ specifies significant change at indicated time comparison, ‘0’, not selected. Two additional worksheets display read counts for all genes (27,179) obtained from total RNA (transcriptome) and polysomal RNA-seq analysis.

**Supplementary Table 2.** Datasets for the GO enrichment analysis with the differentially expressed genes (DEGs) included in the expression profiles shown in Figure 2C. Columns to the right demarcate significant changes in expression (*p*(BH) < 0.05 and abs(FC) > log_2_(1.2)) at the indicated time comparisons with ‘1’; no change ‘0’; T, transcriptome; P, polysome. Separate worksheets are given for the GO enrichment analysis for all 672 DEG, the 76 DEG of the translatome (i.e., polysome) and for each subcluster (C1-C6). GO Categories: BP, Biological Process; CC, Cellular component; MF, Molecular Function. Terms discussed in the main text or displayed in Fig. 2b are labelled in red in the spreadsheet.

**Supplementary Table 3.** Translational efficiency analysis using anota2seq across all paired-age comparisons and expressed genes. The column description is given in the ‘information’ worksheet.

**Supplementary Table 4.** Datasets for the clustering analysis and functional annotation of genes identified with anota2seq as significant in the translation, abundance, and buffering modes. GO enrichment analysis for each subclusters are provided in separate worksheets. GO categories: BP, Biological Process; CC, Cellular component; MF, Molecular Function. Terms displayed in (Figure 3C,E; heatmap cluster) are in red if *p* < 0.01 and FDR < 0.05; in blue, *p* < 0.01 but FDR > 0.05.

**Supplementary Table 5.** SUPPA analysis data file for 1,474 alternative splicing (AS) events allocated to the seven splicing event types. Columns specify the following (from left to right): Splice event type, Locus of splicing event, Gene symbol, Entrez ID, Ensemble ID, Biotype, chromosome location, whether it is a RBM3 target (‘1’) (if not (‘0’), a CIRBP target (‘1’), Refseq annotations, alternative transcript names. The next columns contain dPSI and *p*-values (BH corrected) obtained from transcriptome (Ts) or polysomes (Ps) at the indicated age comparisons. Significant events (*p*(BH) < 0.05) in Ts (orange) and Ps (blue) are marked with ‘1’. Last columns list the replicate PSI values for Ts and Ps at indicated ages that were used to calculate dPSI values. The last column provides a link to the UCSC genome browser.

**Supplementary Table 6.** GO enrichment analysis of AS genes identified with SUPPA2 in pairwise age comparisons. The ‘Summary’ worksheet depicts the number of events/genes identified across all paired-age comparisons (*p*(BH) < 0.05). Three different worksheets display GO terms at indicated age comparisons (left columns) for i) ‘all’ selected transcripts, ii) transcripts in the transcriptome and iii) in the translatome (polysomes). Terms with FDR < 0.05 are indicated with red ink; terms highlighted in green are depicted in Figure 5C or discussed in the text. GO categories: BP, Biological Process; CC, Cellular Component; MF, Molecular Function.

**Supplementary Table 7.** LABRAT analysis for evaluation of alternative poly(A) site selection across age comparisons. A description of all information for the various spreadsheets is given in the ‘info’ sheet. Genes labelled in red ink are selected with FDR < 0.05.
