# Supplementary material for: A post-transcriptional regulatory landscape of aging in the female mouse hippocampus
Source: Front Aging Neurosci. 2023 Mar 24;15:1119873. doi: 10.3389/fnagi.2023.1119873 (PMC10135431; doi:10.3389/fnagi.2023.1119873)
Supplement: Supplementary file 9 [file Data_Sheet_2.PDF]

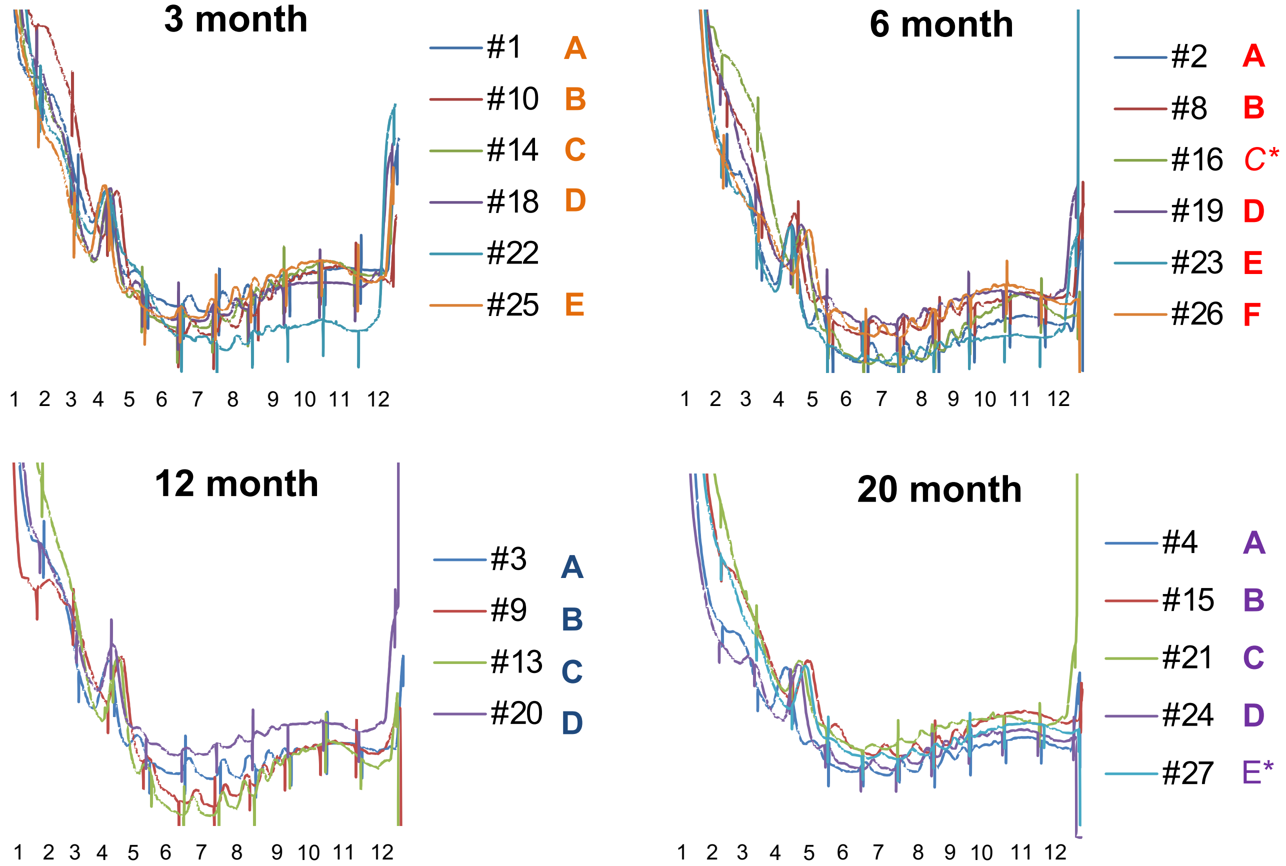

Supplementary Figure 1. Polysomal profiles of female mice hippocampi at indicated ages. Letters on the right hand-side correspond to the process sample number referring to RNA-seq samples; the letter code relates to sample labels for PCA analysis (Supplementary Figure 2). Samples with a star (*) were removed after batch-correction. Fraction numbers are indicated at the bottome; factions 7-12 were collected for RNA-seq representing the translatome.

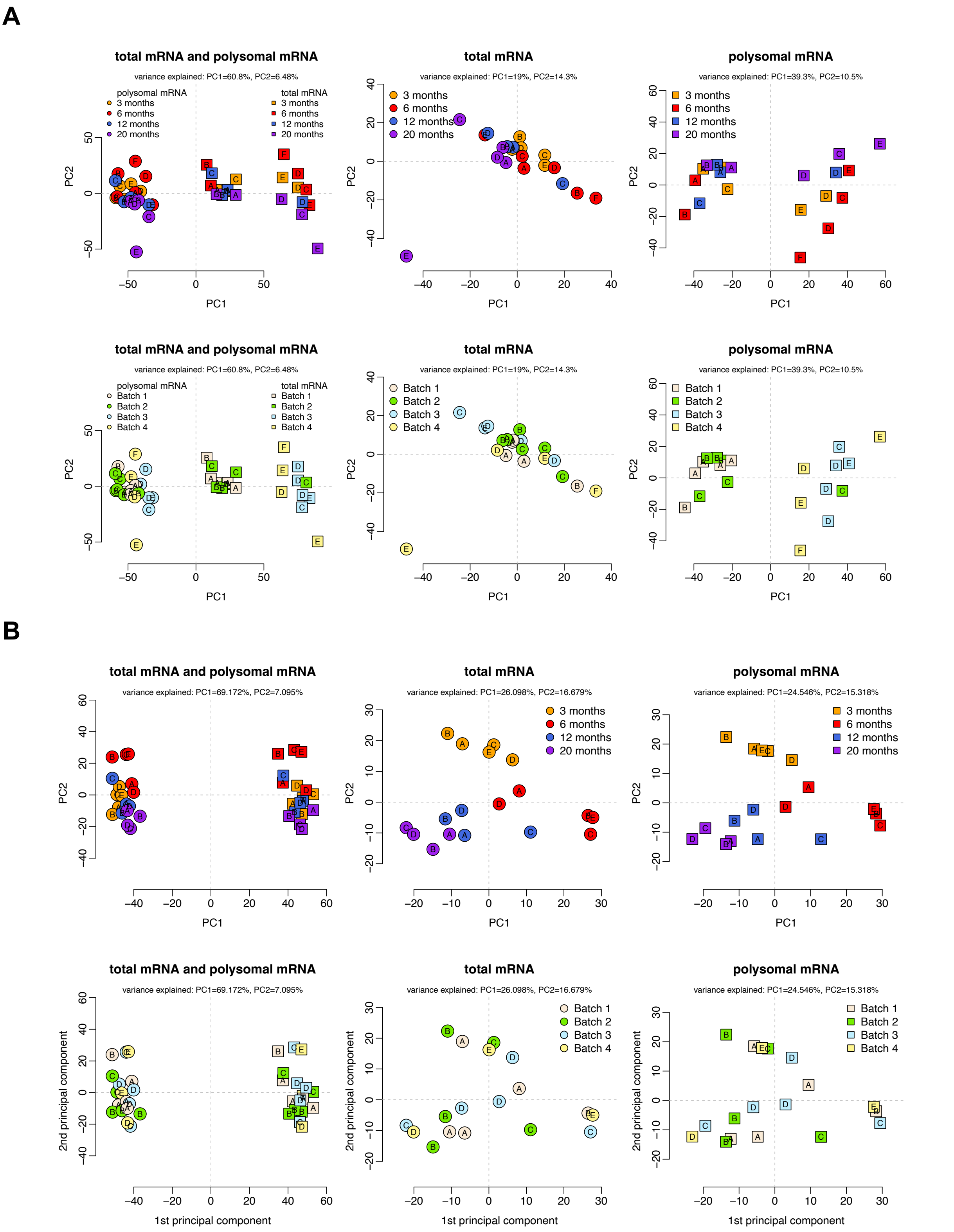

Supplementary Figure 2. Batch correction and PCA analysis of RNAseq samples. **(A)** PCA of all original samples (n=40; left column), total mRNA samples (n=20, central column), and polysomal mRNA samples (n=20, right column). **(B)** PCA after outlier removal and batch correction of all samples (n=36; left column), total mRNA samples (n=18, central column), and polysomal mRNA samples (n=18, right column). Samples are coloured by age (top row) and by batch (bottom row). Circles identify polysomal mRNA and squares correspond to total mRNA samples. Replicates are indicated with letters A to F.

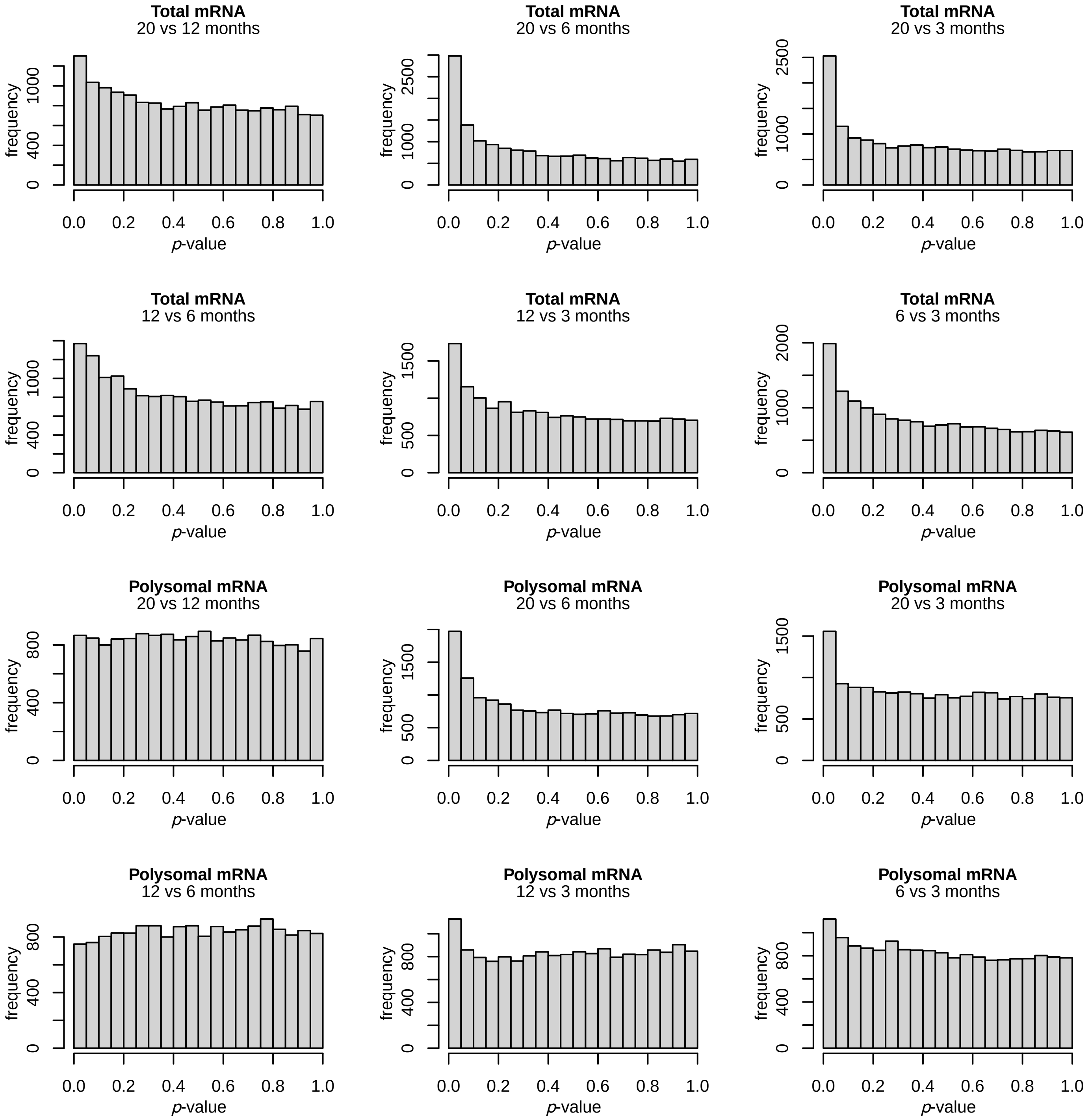

Supplementary Figure 3. Distribution of *p*-values from all age pairwise contrasts in total mRNA and in polysomal mRNAs. Differential expression was evaluated with the edgeR quasi-likelihood pipeline for 16,801 expressed genes.

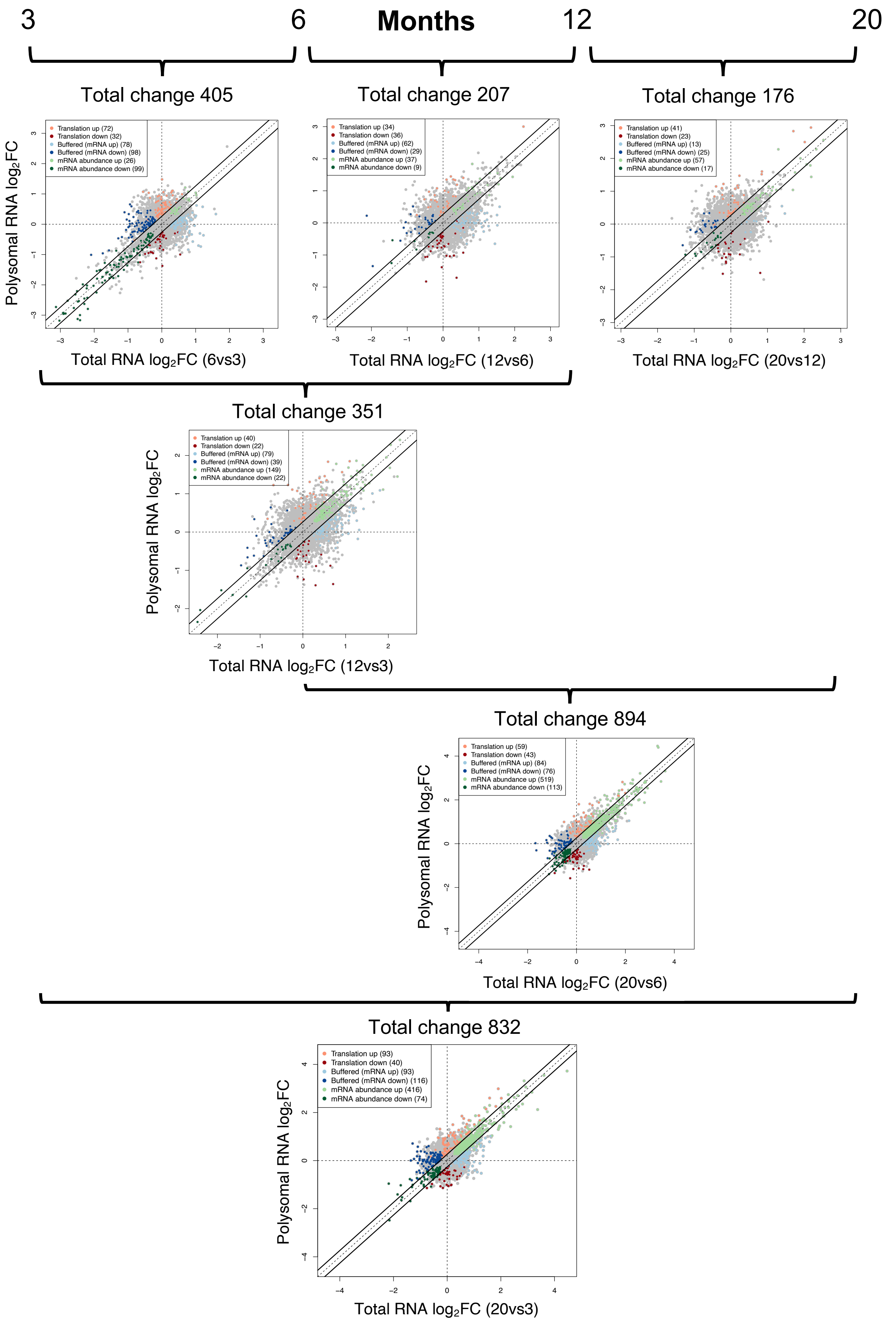

Supplementary Figure 4. Relative FC of transcript levels in the transcriptome (total RNA) and translatome (polysomal RNA) in paired-age comparisons identified with anota2seq. Genes allocated to the indicated regulatory modes are depicted with different colors. The number of genes in each mode is indicated within brackets.

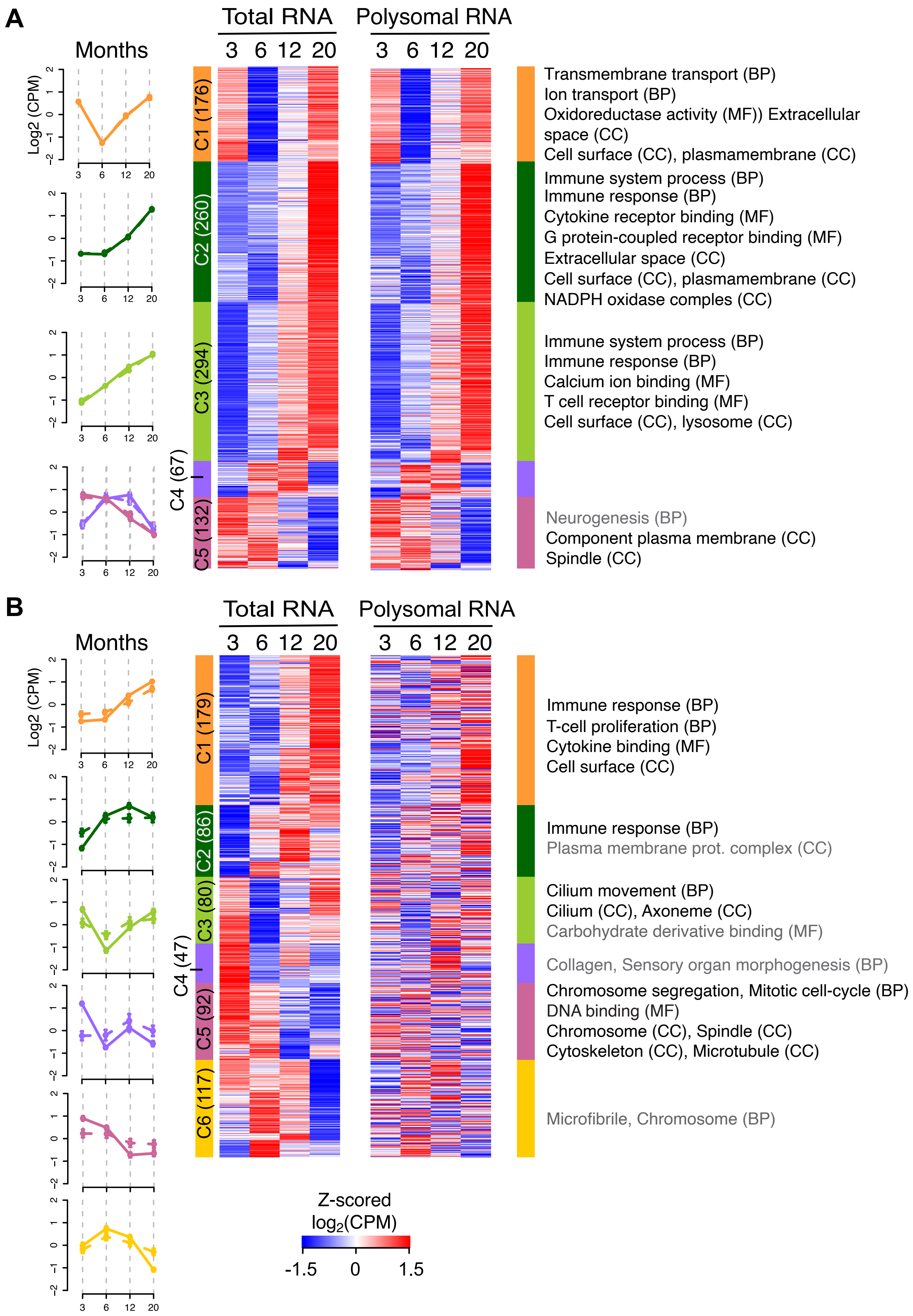

Supplementary Figure 5. Clusters of gene expression profiles identified with anota2seq. Heatmap and averaged temporal profiles of genes identified in **(A)** ‘abundance’ mode (929 genes) and **(B)** ‘buffering’ mode (601 genes) during aging. Clustering was based on total RNA (transcriptome) temporal profiles (left), and corresponding translatome data are shown to the right. The average profiles for each cluster are shown to the left with a continuous line for total RNA, a dashed line for polysomal RNA and error bars showing 95% CI. To the right, significantly enriched GO terms in each cluster. GO categories: BP, biological process, CC, cellular compartment, MF, molecular function. In black, $p < 0.01$ and FDR < 0.05; in grey, $p < 0.01$ but FDR > 0.05.

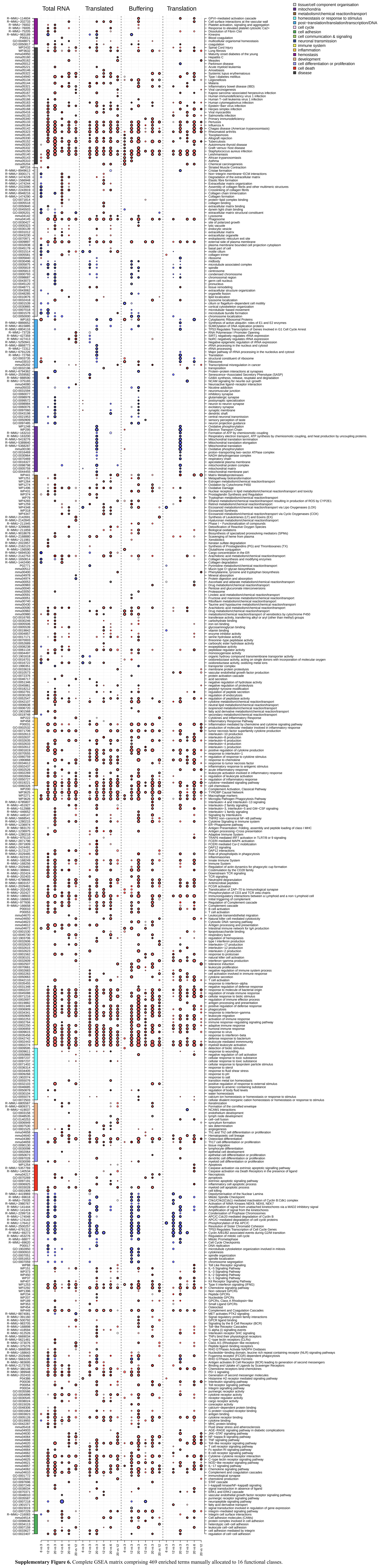

Supplementary Figure 6. Complete GSEA matrix comprising 469 enriched terms manually allocated to 16 functional classes.

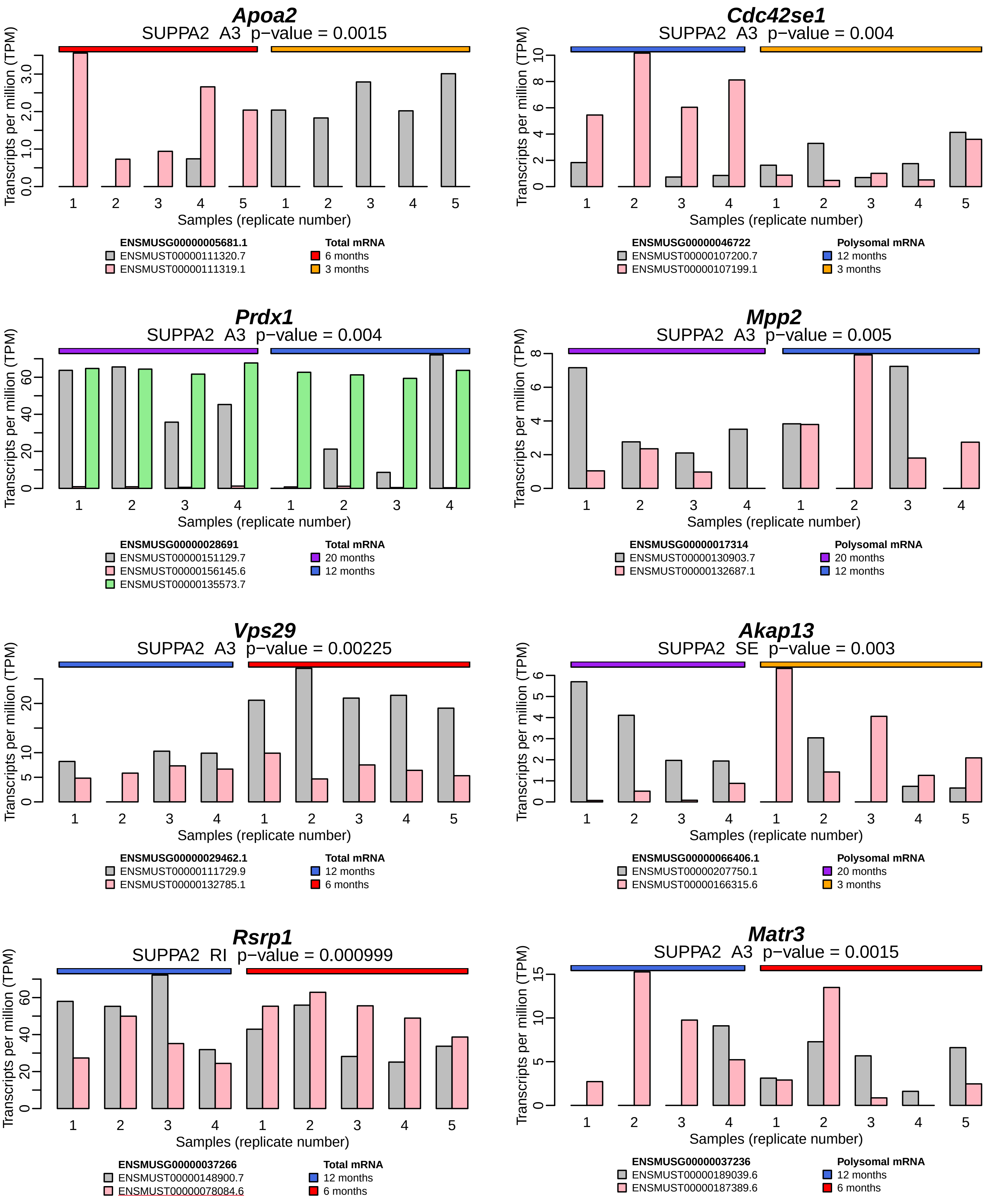

Supplementary Figure 7. Examples of AS events identified with SUPPA2. Bar plots show the expression level of the indicated gene transcripts (colored bars with ENSEMBL accession numbers) across all replicate RNA-seq samples for the indicated age groups. The AS event type and *p*-value of the test for differential relative inclusion values across age groups are displayed below the gene symbol. Transcriptome (total RNA) events are shown to the left; translatome (polysomal mRNA) events to the right.

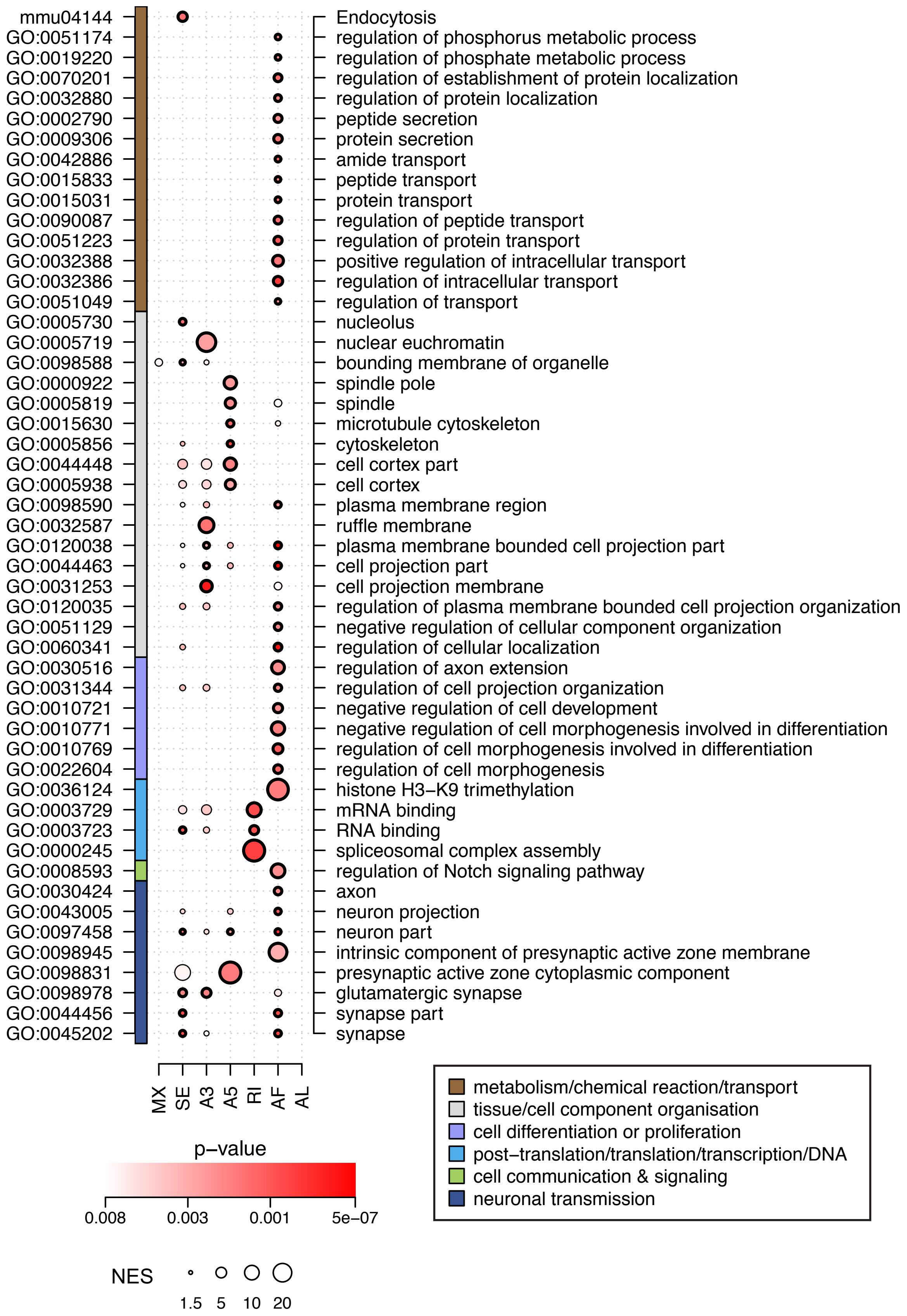

Supplementary Figure 8. Display of all 51 themes overrepresented across the seven splicing event types (extension of Figure 6C). Functional themes were identified among all genes of the indicated splicing category across all age comparisons and selected with $p < 0.01$, FDR < 0.05. The diameter of the circle is proportional to the NES score, the colormap refers to the p-value, circles in bold refer to selected FDR < 0.05.

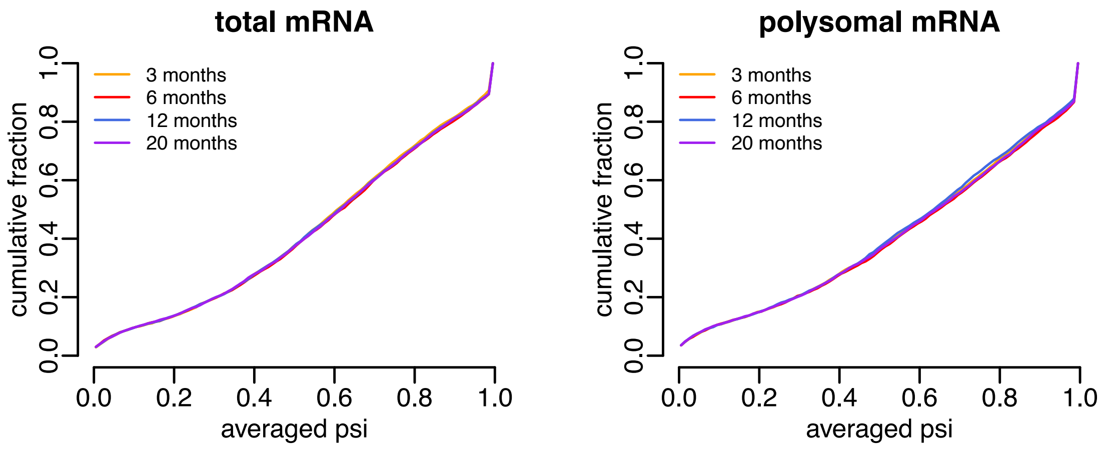

Supplementary Figure 9. Alternative polyadenylation (APA) site usage across four ages in the hippocampus. Cumulative frequency plot representing the cumulative fraction of genes (*y*-axis) with averaged psi values (*x*-axis) at the four different ages (colored lines). Transcriptome (left, n = 4,912) and translatome (right, n = 4,714). The psi value denotes the fraction of distal *vs*. proximal APA site usage; psi = 0 indicates exclusive usage of proximal APA, psi = 1 of distal APA site.
